# Increased co-expression of PD1 and TIM3 is associated with poor prognosis and immune microenvironment heterogeneity in gallbladder cancer

**DOI:** 10.1186/s12967-023-04589-3

**Published:** 2023-10-12

**Authors:** Xing He, Yaorong Peng, Gui He, Huilin Ye, Liqiang Liu, Qixian Zhou, Juanyi Shi, Sha Fu, Jie Wang, Zhenyu Zhou, Wenbin Li

**Affiliations:** 1 Department of Biliary and Pancreatic Surgery, Sun Yat-Sen Memorial Hospital, Sun Yat-Sen University, No.107 Yanjiang West Road, Yuexiu District, Guangzhou, 510120 Guangdong People’s Republic of China; 2 Department of Hepatobiliary Surgery, Sun Yat-Sen Memorial Hospital, Sun Yat-Sen University, Guangzhou, 510120 People’s Republic of China; 3 Guangdong Provincial Key Laboratory of Malignant Tumor Epigenetics and Gene Regulation, Sun Yat-Sen Memorial Hospital, Sun Yat-Sen University, Guangzhou, 510120 People’s Republic of China; 4 Cellular & Molecular Diagnostics Center, Sun Yat-Sen Memorial Hospital, Sun Yat-Sen University, Guangzhou, 510120 People’s Republic of China

**Keywords:** Tumor microenvironment, Tumor-infiltrating lymphocytes, Immune checkpoint inhibitors, Tumor biomarkers

## Abstract

**Background:**

The effectiveness of immune checkpoint inhibitors in treating gallbladder cancer (GBC) remains unsatisfactory. Recently, several new immune checkpoints have been identified. However, investigations exploring these immune checkpoints in GBC are limited. In this study, we aim to investigate the expression patterns and clinical implications of various immune checkpoints, and further characterize the spatial and quantitative heterogeneity of immune components in GBC.

**Methods:**

We employed single and multiplex immunohistochemistry to evaluate the expression of five immune checkpoint markers and four immune cell markers in the primary tumor core, hepatic invasion margin, and liver metastasis. Subsequently, we analyzed their interrelationships and their prognostic significance.

**Results:**

We observed a robust positive correlation between PD1/TIM3 expression in GBC (R = 0.614, P < 0.001). The co-expression of PD1/TIM3 exhibited a synergistic effect in predicting poor prognosis among postoperative GBC patients. Further analysis revealed that the prognostic significance of PD1/TIM3 was prominent in the subgroup with high infiltration of CD8 + T cells (P < 0.001). Multiplex immunohistochemistry reveals that PD1 + TIM3 + FOXP3 + cells constitute a significant proportion of FOXP3 + TILs in GBC tissue. Moreover, the co-high expression of PD1 and TIM3 is positively correlated with the accumulation of CD8 + TILs at the hepatic invasion margin. Lastly, our findings indicated reduced expression levels of immune checkpoints and diminished immune cell infiltration in liver metastases compared to primary tumors.

**Conclusions:**

Increased co-expression of PD1/TIM3 is associated with poor prognosis in GBC patients and is related to the heterogeneity of immune microenvironment between GBC primary tumor and its hepatic invasion margin or liver metastases, which may be a potential target for future immunotherapy of GBC.

**Supplementary Information:**

The online version contains supplementary material available at 10.1186/s12967-023-04589-3.

## Introduction

Gallbladder cancer (GBC) is the most commonly diagnosed malignant tumor of the biliary system. Its incidence has been increasing in recent years in the Asian region, including China [1]. GBC is highly malignant and often insidious in its early stages, with a poor prognosis compared to other common gastrointestinal malignancies [2]. Due to the suboptimal nature of existing treatment approaches for GBC, early surgical intervention remains the most effective approach [3]. Hence, it is imperative to investigate novel therapeutic regimen to enhance the treatment options accessible for patients with GBC.

Immunotherapy plays a crucial role in disrupting the immune microenvironment of tumors, overcoming tumor-specific suppression, and stimulating the body's immune response to eliminate tumors through immune-mediated mechanisms [4]. The clinical application of immune checkpoint inhibitors (ICIs) targeting co-inhibitory receptors such as CTLA4 and PD1 has revolutionized the treatment of malignant tumors and significantly improved the prognosis of patients with advanced malignancies such as melanoma and non-small cell lung cancer [5]. It is noteworthy that ongoing clinical trials are presently examining the combination of monoclonal antibodies targeting additional immune checkpoints, including TIM3, LAG3, and TIGIT, in conjunction with PD-1 inhibitors [6–9]. Taking TIM3 as an example, research has revealed that the combined application of PD1 and TIM3 inhibitors significantly enhances the immune activity of effector T cells in animal models such as acute myelogenous leukemia and lung cancer, surpassing the efficacy of using PD1 inhibitors alone [10, 11]. However, research concerning the combined blockade of immune checkpoints in GBC remains limited.

Advances in the study of biomarkers for ICIs have unveiled the intricate heterogeneity within the tumor immune microenvironment, which underscores the critical significance of investigating variations in the quantity and distribution of immune components [12]. Notably, the quantification of PD-L1 expression through immunohistochemistry has been approved as a robust biomarker for selecting PD1/PD-L1 inhibitors [13]. Furthermore, researchers [14] have categorized patients into four distinct subtypes of tumor immune microenvironments based on PD-L1 expression and immune cell infiltration to help design immunotherapy strategies. Thus, it is intriguing to explore the expression of diverse immune checkpoint proteins and their correlation with immune cell infiltration in GBC [15].

In this study, we employed single immunohistochemistry and multiplex fluorescence immunohistochemistry to explore the expression of five immune checkpoints within GBC tissues. The correlation among these immune checkpoints and their relationship with clinical characteristics and prognosis were also assessed. As the results show the correlation between PD1 and TIM3 and its importance for prognosis, we assessed how their expression patterns correlated with the density of immune cells and explored their co-localization on cells. Finally, we explored the relationship between the co-high expression of PD1/TIM3 and the heterogeneity of the immune microenvironment within the primary tumor core, hepatic invasion margin, and liver metastasis.

## Methods and materials

### Patients and samples

The retrospective study was conducted on a cohort comprising 127 patients who were diagnosed with gallbladder cancer and underwent surgical resection at Sun Yat-Sen Memorial Hospital, Sun Yat-Sen University between 2013 and 2020. Among these patients, 51 cases had concomitant liver invasion (i.e. pathologically confirmed direct invasion of liver tissue by the primary gallbladder cancer tumor) and 21 cases had concomitant liver metastases (i.e. distant intrahepatic metastases of the tumor). Inclusion criteria for the cohort were as follows: [1] postoperative histopathological confirmation of GBC with available formalin-fixed, paraffin-embedded tissue samples; [2] no history of preoperative anticancer treatment; [3] the availability of complete histopathological and follow-up information. Patients who died perioperatively, had a history of autoimmune disease or other malignancies, or had severe cardiac, pulmonary or other systemic diseases were excluded from the cohort. The observed endpoint of follow-up was overall postoperative survival time. Postoperative overall survival (OS) was defined as the time interval between the date of surgery and the date of death. In cases where patients remained alive at the conclusion of the study, their follow-up time was recorded as their latest follow-up visit. The most recent follow-up visit for all patients occurred in October 2022.

### Immunohistochemistry (IHC)

Tissue Sects. (4 μm) were prepared by FFPE obtained as described previously, and the sections were dewaxed by employing xylene and hydrated using a gradient concentration of ethanol. Subsequently, the sections underwent high temperature and high-pressure antigen retrieval in EDTA buffer (pH 9.0), followed by endogenous peroxidase inactivation (3% hydrogen peroxide, 10 min) and serum blocking (goat serum, 30 min). The sections were subsequently incubated overnight at 4 °C with the designated primary antibody relevant to the study (refer to Additional file 1: Table S1 for antibody details). Following primary antibody incubation, the slides were subsequently incubated with the appropriate horseradish peroxidase (HRP)-conjugated secondary antibody and visualized utilizing the DAB solution. Subsequently, the slides were counterstained with hematoxylin, dehydrated, and finally sealed. Finally, they were scanned using a fully automated sweeper (Unic-med). A positive control (tonsil tissue) and a negative control (using PBS buffer instead of the primary antibody) were set up for each staining experiment to ensure quality control of the staining.

### Quantification of immune checkpoints expression

The expression of immune checkpoints (including PD1, TIM3, LAG3, TIGIT, CTLA4) was analyzed using *QuPath* (https://qupath.github.io) [16, 17]. The detailed quantitative protocol is listed in Additional file 2. Briefly, a physician with specialized training in pathology determined the software parameters based on experimental controls to optimize the detection indices. These indices included staining intensity (0- no staining, 1 weak, 2- moderate, 3- strong) and the percentage of positive cells. Immune checkpoint expression was quantified by multiplying the values of staining intensity and percentage of positive immune cells to obtain an H-score ranging from 0 to 300. As a quality control measure, all analyzed data underwent a secondary review by a pathologist before being exported. All analytical assessments were blinded to the maximum practical extent.

### Assessment of immune cell counts

Unlike the immune checkpoint staining score, the immune cell staining score focused on assessing the density of local immune cell infiltration within the tumor section, rather than considering the overall tumor landscape. Building upon previous methodologies [18], We quantified the counts of CD4 + , CD8 + , Foxp3 + , and CD68 + cells in both the tumor core and the hepatic invasion margin (defined as the 500 μm wide area on both sides of the invasive border [19, 20]) using *QuPath* (https://qupath.github.io) [17]. The detailed quantitative protocol is listed in Additional file 2. Briefly, pathologists utilized software to identify four ‘‘hot spots’’ within the annotation, each measuring 400 μm × 400 μm and containing the highest number of positive cells. The final cell density data was derived from the average counts of these four “hot spots”. All analytical assessments were blinded to the maximum practical extent.

### Cut-off values of continuous variables

We used *X-TILE*, as described in previous studies [21], to determine the optimal cut-off values for immune checkpoint expression score and immune cell infiltration density. *X-TILE* is a software program specifically designed to determine the optimal cutoff points for biomarkers in survival analysis. It considers continuous variables, survival time, and survival status, utilizing either the minimum p-value or the maximum chi-square value as criteria. Using these cutoff values, the patient cohort was divided into two separate groups for further statistical analysis. The cutoff values for H score and cell density mentioned in this article are shown in Additional file 1: Table S2.

### Multiplex immunohistochemistry (mIHC)

For fluorescence multiplex immunohistochemistry analysis, a five-color fluorescence kit (Absin Bioscience, abs50013) based on tyramine signal amplification (TSA) was used according to the manufacturer's protocol. The procedure involved dewaxing, hydration, antigen retrieval, quenching of endogenous peroxidase, and serum blocking as described for IHC. Tissue sections were subsequently subjected to two to four consecutive cycles of incubation with the primary antibodies listed in Additional file 1: Table S1, with each cycle lasting 1 h at room temperature, based on the specific requirements of the study. Following this, secondary antibodies and TSA solutions were applied. TSA dyes 520, 570, 620, and 700 were used for staining. After the final TSA staining cycle, nuclei were counterstained with DAPI for 5 min. Sections were then sealed using an anti-fluorescence quenching agent. The sections were then scanned for fluorescence panoramic images using the Vectra Polaris fully automated quantitative pathology imaging system instrument (Akoya Biosciences) and analyzed by a pathologist using INFO software (version 2.4.2, Akoya Biosciences). The detailed assessment protocol is listed in Additional file 2. In brief, following tissue and cell segmentation, cell subgroups were identified using markers through the phenotype tool available in the INFO.

### Statistical analysis

Survival curves depicting postoperative OS were constructed using the Kaplan–Meier method, and the log-rank test was employed to assess survival differences between groups. The correlation between H-scores at each immune checkpoint was analyzed using either Pearson's or Spearman's correlation coefficient. The association between PD-1 and TIM3 expression and clinicopathologic characteristics was evaluated using either the chi-squared test or Fisher's exact test. The t-test or Wilcoxon-Mann–Whitney test was utilized for comparing two independent samples, while the t-test or Wilcoxon’s matched signed rank test was employed for comparing paired samples. Univariate and multifactorial analyses were conducted using the Cox proportional hazards regression model. All statistical analyses were performed using SPSS (version 25) or R software (version 4.2.1). Statistical significance was defined as *P* < 0.05.

## Results

### Clinicopathological characteristics of GBC cohort and specimens

Clinicopathological characteristics of the study cohort of patients with GBC are listed in Additional file 1: Table S3. The study enrolled 127 patients with a median age of 63 years (range: 33–88 years). Patients were staged in accordance with the AJCC TNM eighth edition, with 7.9% in stage I, 18.1% in II, 20.5% in III, and 53.5% in IV. All cases had complete histopathologic data, including differentiation and microvascular invasion, and were diagnosed as GBC by clinical pathologists. When follow-up ends, 72.4% (92/127) had died of gallbladder cancer. The median postoperative overall survival was 15 months, with survival rates of 55.9%, 29.4%, and 22.3% at 1, 3, and 5 years, respectively. In this study cohort, 51 patients had concomitant liver invasion and 21 patients had concomitant liver metastases.

### Expression landscape of immune checkpoints in GBC

**Fig. 1:**
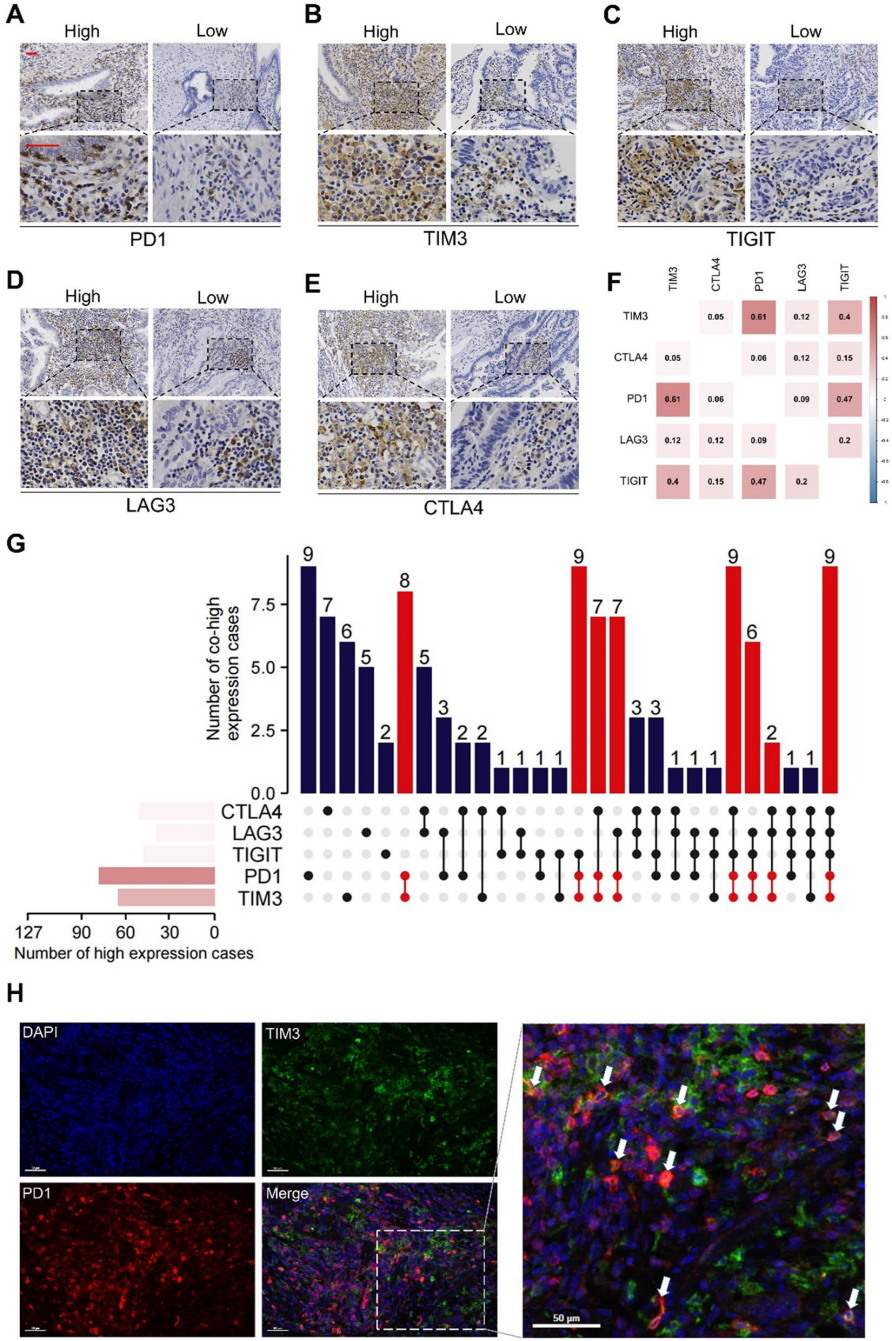
Expression of immune checkpoints in tumor tissues from GBC patients. **A–E**, Representative IHC images of PD1, TIM3, TIGIT, LAG3, and CTLA4 expression within the tumor (Red scale bar is 50 μm). **F**, The correlation matrix of the expression H-score of immune checkpoints, and the coefficients marked in boxes indicate the correlation between a pair of immune checkpoints. **G**, Upset plot visualizing the co-expression profiles of ICPs in GBC. **H**, Representative mIHC images of PD1 and TIM3. The nucleus is labeled with DAPI (blue), and White arrows indicate the colocation of PD1 and TIM3

We explored the expression of PD1, TIM3, TIGIT, LAG3, and CTLA4 in GBC tissues. Representative IHC images illustrating the staining of these immune checkpoints in GBC tissues are presented in Fig. 1A–E. These immune checkpoints were mainly stained on cell membrane and cytoplasm. Correlation analysis of the H-scores for the immune checkpoints revealed significant positive associations between TIM3 and PD1 expression (R = 0.614, *P* < 0.001), PD1 and TIGIT expression (R = 0.466, *P* < 0.001), and TIM3 and TIGIT expression (R = 0.401, *P* < 0.001) **(**Fig. 1F**)**. To determine optimal expression cut-off values for each immune checkpoint, we employed *X-tile* software. Subsequently, the patient cohort was divided into high and low expression subgroups. The correlation between the expression of immune checkpoint and clinicopathological features is presented in Table 1 and Additional file 1: Table S4. The expression of PD1 demonstrated significant correlations with T-stage (*P* = 0.029), N-stage (*P* = 0.002), M-stage (*P* = 0.030), and the degree of histologic differentiation (*P* = 0.039). The expression of TIM3 was correlated with N-stage (*P* = 0.053). The immune checkpoint expression profile (Fig. 1G) was calculated, indicating that PD1, TIM3, TIGIT, LAG3, and CTLA4 were highly expressed in 61.4% (78/127), 53.5% (68/127), 38.6% (49/127), 36.2% (46/127), and 41.7% (53/127) of cases, respectively. Notably, co-high expression of PD1 and TIM3 was the most frequently observed, accounting for 44.9% of cases (57/127). The co-high expression of PD1 and TIM3 correlated with more advanced N stage (*P* = 0.013), more advanced TNM stage (*P* = 0.075), and poorer histologic differentiation (*P* = 0.061) (Table 1). Furthermore, multiplex immunohistochemistry staining revealed a widespread colocalization of PD1 and TIM3 on the same cells in GBC (Fig. 1H). Thus, these findings highlight the expression landscape of the five immune checkpoints in GBC, particularly the highly positive correlation between PD1 and TIM3, which motivated further investigation into their clinical significance.

**Table 1:** The correlation between PD1, TIM3 and PD1/TIM3 expression status and clinicopathological characteristics in GBC

Characteristic		PD1 expression			TIM3 expression			PD1/TIM3 co-high expression		
	Cases.n	High	Low	P value	High	Low	P value	Present	Absent	P value
Gender				0.706			0.954			0.689
Female	61	39	22		32	29		29	32	
Male	66	39	27		36	30		28	38	
Age				0.913			0.934			0.452
< 60 years	50	31	19		27	23		25	25	
> 60 years	77	47	30		41	36		32	45	
Tumor size				0.813			0.824			0.878
> 3 cm	67	40	27		37	30		31	36	
< 3 cm	60	38	22		31	29		26	34	
Microvascular invasion				0.835			0.955			0.953
no	75	45	30		40	35		33	42	
yes	52	33	19		28	24		24	28	
T				**0.029**			0.962			0.243
Low(1.2)	46	22	24		24	22		17	29	
High(3.4)	81	56	25		44	37		40	41	
N				**0.002**			**0.053**			**0.013**
Low(0)	52	23	29		22	30		16	36	
High(1.2)	75	55	20		46	29		41	34	
M				**0.030**			0.893			0.274
Absent(0)	94	52	42		50	44		39	55	
Present(1)	33	26	7		18	15		18	15	
TNM stage				**0.014**			0.270			0.075
Low(I.II.III)	59	29	30		28	31		21	38	
High(IV)	68	49	19		40	28		36	32	
Histologic differentiation				**0.039**			0.186			0.061
Moderate. Well	75	40	35		36	39		28	47	
Poor	52	38	14		32	20		29	23	
CA19-9				0.539			0.882			0.791
Negative	54	31	23		28	26		23	31	
Positive	73	47	26		40	33		34	39	
CEA				0.275			0.880			0.627
Negative	82	47	35		43	39		35	47	
Positive	45	31	14		25	20		22	23	

Bold values indicates statistically significant

### PD1 and TIM3 synergistically predict poor prognosis in GBC patient

**Fig. 2:**
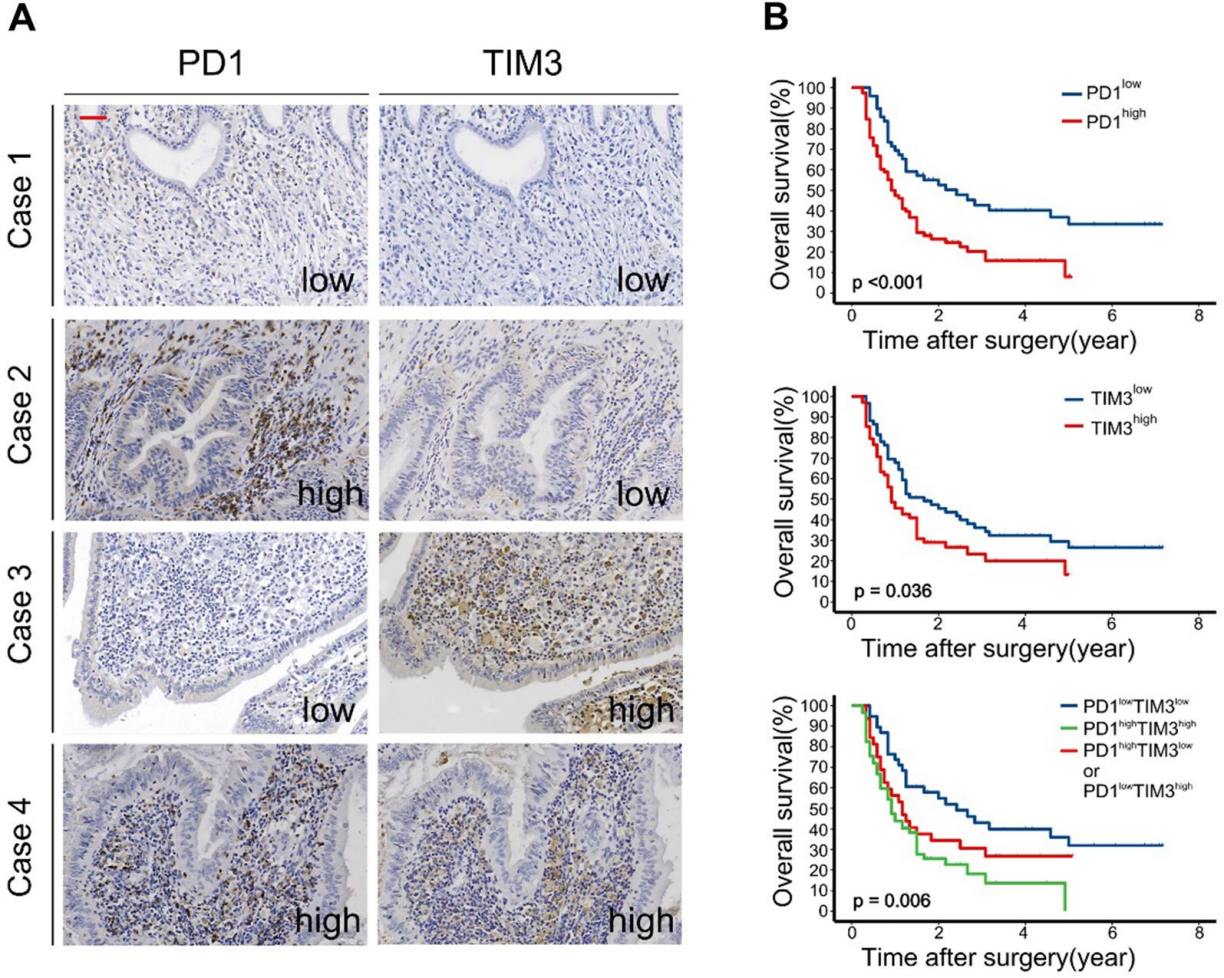
Increased co-expression of PD1 and TIM3 predicted poor OS of patients with GBC **A,** Representative IHC images of four cases are presented based on the expression status of PD1 and TIM3 (Red scale bar is 100 μm). **B**, Kaplan–Meier survival curves for postoperative OS of GBC patients according to PD1 and TIM3 expression status

Kaplan–Meier (K-M) survival analysis showed that high expression of PD1 or TIM3 was significantly associated with a worse postoperative OS in GBC patients (Fig. 2B). On the other hand, although patients with high expression of TIGIT, LAG3, or CTLA4 had slightly shorter OS, no statistical significance was observed (Additional file 1: Figure S1). Considering the highly positive correlation between PD1 and TIM3 expression and their prognostic significance, patients were classified into four subgroups (Fig. 2A shows representative cases from each of these four groups) based on the co-high expression pattern, and K-M survival analysis was performed. The results showed that patients in the PD1^high^TIM3^high^ group had the worst prognosis, either PD1 or TIM3 low expression partially improved the poor prognosis, while patients with PD1^low^TIM3^low^ had the best prognosis (Fig. 2B). Univariate regression analysis showed that TNM stage IV, R1 resection, and poor differentiation were significant predictors of shorter OS (Table 2). Moreover, both high expression of PD1 or TIM3 alone, as well as co-high expression of PD1 and TIM3, were significantly associated with shorter OS. Multivariate regression analysis was then performed, taking into account the clinical relevance of adjuvant therapy in GBC, despite its lack of prognostic significance in univariate regression analysis. The results demonstrated that TNM stage, surgical margin, adjuvant therapy, and tumor differentiation were independent predictors of OS in GBC patients. Unexpectedly, the predictive value of PD1 or TIM3 alone was not retained in the multivariable analysis (multivariable analysis a). To address this, another multivariable analysis (multivariable analysis b) was performed, revealing that PD1^high^TIM3^high^ represented an independent risk factor for OS, indicating that the co-high expression of PD1 and TIM3 exerted a synergistic effect in predicting the prognosis of GBC patients, surpassing the predictive value of PD1 or TIM3 alone (Table 2). Collectively, these data strongly suggest that the co-high expression of PD1 and TIM3 serves as an adverse prognostic factor in GBC.

**Table 2:** Univariate and multivariate regression analysis of prognostic factors correlated with postoperative OS

	Univariable analysis		Multivariable analysis^a^		Multivariable analysis^b^		
Variables	HR (95%CI)	P value	HR (95%CI)	P value		HR (95%CI)	P value
Gender							
Female	Reference						
Male	1.10 (0.73 to 1.66)	0.640					
Age							
< 60 years	Reference						
> 60 years	1.30 (0.85 to 1.99)	0.223					
Gall stone							
Absent	Reference						
Present	1.36 (0.91 to 2.06)	0.137					
Tumor size							
< 3 cm	Reference						
> 3 cm	1.15 (0.76 to 1.73)	0.513					
TNM stage							
Low (I.II. III)	Reference		Reference			Reference	
High(IV)	6.11 (3.84 to 9.72)	** < 0.001**	4.04 (2.07to7.87)	** < 0.001**		4.14 (2.12 to 8.09)	** < 0.001**
Surgical margin							
R0	0.16 (0.10 to 0.25)	** < 0.001**	0.51 (0.28to0.92)	**0.026**		0.51 (0.28 to 0.92)	**0.025**
R1	Reference		Reference			Reference	
Adjuvant therapy							
Present	1.14 (0.74 to 1.73)	0.555	0.58 (0.36to0.92)	**0.021**		0.59 (0.37 to 0.94)	**0.027**
Absent	Reference		Reference			Reference	
Histologic differentiation							
Moderate. Well	Reference		Reference			Reference	
Poor	7.04 (4.36 to 11.36)	** < 0.001**	5.06 (3.00to8.55)	** < 0.001**		5.05 (3.00 to 8.53)	** < 0.001**
TIM3							
Low	Reference		Reference				
High	1.57 (1.03 to 2.38)	**0.036**	1.16 (0.70to1.91)	0.561			
PD1							
Low	Reference		Reference				
High	2.15 (1.37 to 3.36)	** < 0.001**	1.54 (0.91to2.62)	0.111			
GROUP							
GROUPI	Reference					Reference	
GROUPII	1.56 (0.88 to 2.77)	0.13				1.54 (0.86 to 2.76)	0.142
GROUPIII	2.27 (1.36 to 3.77)	**0.002**				1.80 (1.08 to 3.02)	**0.025**

GROUPI,PD1low/TIM3low; GROUPII, PD1high/TIM3low or PD1low/TIM3high; GROUPIII, PD1high/TIM3high

Bold values indicates statistically significant

Multivariable analysis ^a^: PD1, TIM3 and other significant clinicopathological characteristics

Multivariable analysis ^b^: GROUP and other significant clinicopathological characteristics

### PD1 and TIM3 widely co-express on Foxp3 + TIL in GBC

**Fig. 3:**
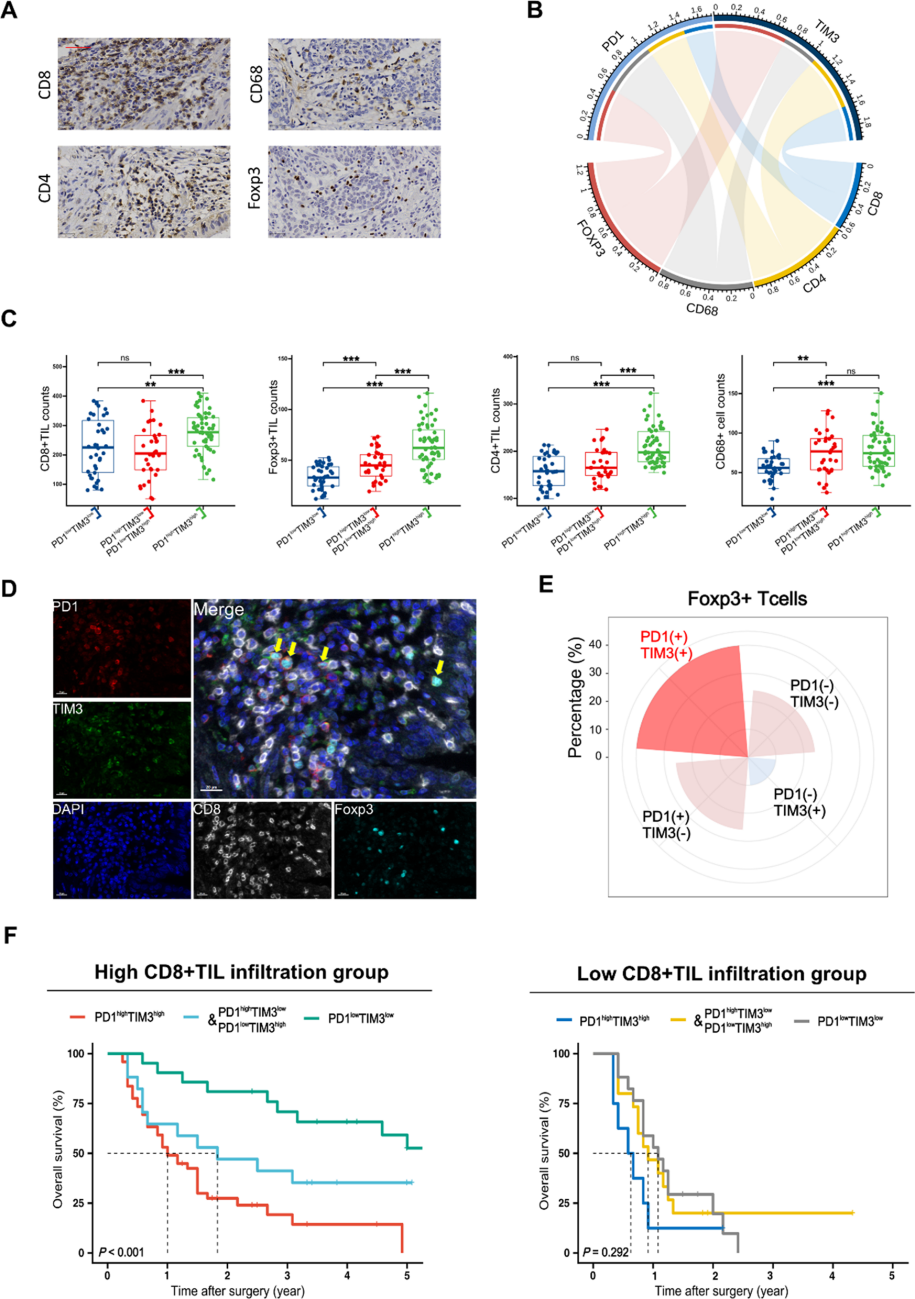
The correlation between the expression of PD1/TIM3 and the infiltration density of immune cells. **A**, Representative IHC images of CD8, CD4, CD68, and Foxp3 staining within the tumor (Red scale bar is 50 μm). **B**, The chord diagram shows the correlation network between immune checkpoints H score and immune cell density in GBC tissue. The band represents a positive correlation between the ICP and immune cell density, and the width indicates the magnitude of Pearson’s correlation coefficient (the P value for testing the correlation coefficient was < 0.05). **C**, The correlation of PD1/TIM3 co-expression status and CD8 + TIL, CD4 + TIL, CD68 + cell, Foxp3 + TIL density in GBC tissue (*P < 0.05; **P < 0.01; ***P < 0.001; ns: no significance). **D**, Representative mIHC images of PD1, TIM3, CD8, and Foxp3 staining within the tumor (White scale bar is 20 μm), yellow arrows indicate the colocation of PD1 and TIM3 on Foxp3 + TIL. **E**, The percentage of Foxp3 + TIL with different PD1/TIM3 co-expression status in GBC tissues. **F**, Kaplan–Meier survival curves for postoperative OS according to PD1 and TIM3 co-expression status in the high/low CD8 + TIL infiltration subgroup

To investigate the immune cells contributing to the expression of PD1 and TIM3 in the GBC microenvironment, we performed CD8, CD4, Foxp3, and CD68 staining to evaluate the infiltration level of CD4 + TILs, CD8 + TILs, Foxp3 + TILs, and macrophages. Representative IHC images for each marker are presented in Fig. 3A. The correlation among the infiltration density of four immune cell types is depicted in Additional file 1: Figure S2. We further examined the correlation between the H-scores of PD1 and TIM3 and the cell density of CD4 + TILs, CD8 + TILs, Foxp3 + TILs, and macrophages **(**Fig. 3B**)**. The analysis revealed a strong association between Foxp3 + TILs and PD1/TIM3 expression (Foxp3^+^-PD1: R = 0.5157, *P* < 0.001; Foxp3^+^-TIM3: R = 0.7142, *P* < 0.001). Additionally, a modest correlation was found between CD8 + TILs and PD1/TIM3 expression (CD8^+^-PD1: R = 0.2848, *P* = 0.0012; CD8^+^-TIM3: R = 0.3403, *P* < 0.001). Moreover, the box plot shows that high expression of PD1 and TIM3 was associated with increased infiltration of CD8 + TILs, Foxp3 + TILs, CD4 + TILs, and macrophages (Additional file 1: Figure S3). Remarkably, PD1 and TIM3 co-high expression was significantly associated with elevated infiltration of these immune cell subsets **(**Fig. 3C**)**. Furthermore, multiplex immunohistochemistry staining revealed widespread co-expression of PD1 and TIM3 on Foxp3 + TILs **(**Fig. 3D**)**. Quantitative analysis indicated that the proportion of PD1 + TIM3 + Foxp3 + TILs was the highest subpopulation within Foxp3 + TILs (40.67%) **(**Fig. 3E**)**, while the proportion of PD1 + TIM3-CD8 + TILs was the highest subpopulation within CD8 + TILs (32.84%) (Additional file 1: Figure S4). Collectively, our data indicate that increased expression of PD1 and TIM3 in tissues is closely correlated with the infiltration density of FOXP3 + TILs. PD1 and TIM3 widely co-express on Foxp3 + TIL in GBC.

### PD1 and TIM3 co-low expression predict better prognosis in GBC patients with high CD8 + TIL infiltration.

We also paid attention to the impact of immune cell infiltration on the prognosis of GBC patients. Our results showed that GBC patients with high infiltration of Foxp3 + TIL had a significantly shorter OS than those with low infiltration (*P* < 0.001) (Additional file 1: Figure S5). In contrast, patients with high infiltration of CD8 + TIL had a significantly longer OS than those with low infiltration (*P* = 0.002) (Additional file 1: Figure S5). Notably, further analysis demonstrated that within the CD8 + TIL hyperinfiltrated subgroup, patients with co-high expression of PD1 and TIM3 had a significantly shorter OS compared to patients with low expression of PD1/TIM3 (*P* < 0.001) (Fig. 3F). In contrast, within the CD8 + TIL hypoinfiltrated subgroup, the expression levels of PD1 and TIM3 had minimal impact on patient survival (*P* = 0.292) **(**Fig. 3F**)**. These results further underscore the crucial role of PD1 and TIM3 in GBC, particularly in cases with high CD8 + TIL infiltration.

### PD1 and TIM3 high expression is associated with higher CD8 + TIL infiltration in the hepatic invasion margin

**Fig. 4:**
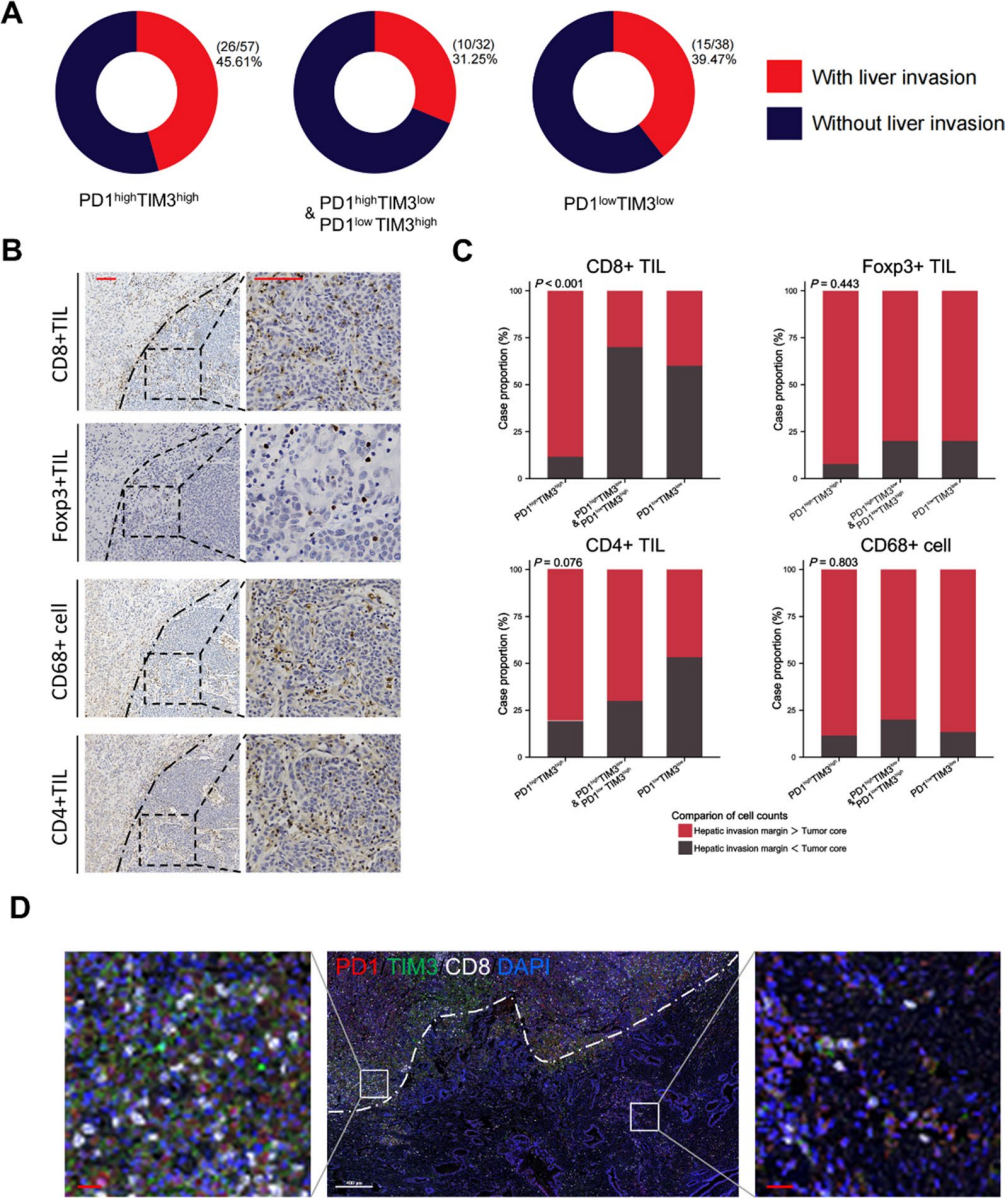
Increased co-expression of PD1 and TIM3 is associated with elevated infiltration density of immune cell at the hepatic invasion margin (HIM). **A**, The donut plots show the proportion of cases with liver invasion in each subgroup. These subgroups were grouped according to PD1/TIM3 expression status. **B**, Representative IHC images of CD8, CD4, CD68, and Foxp3 staining within the hepatic invasion margin (Red scale bar is 50 μm). **C**, Bar plots show that in the PD1/TIM3 co-expression subgroup, more patients have higher counts of immune cells at the hepatic invasion margin than the tumor core. **D**, Representative mIHC images of PD1, TIM3, and CD8 staining on the Hepatic invasion margin (White scale bar is 400 μm; Red scale bar is 30 μm; White dotted line marks the invasive border)

Liver invasion represents a common occurrence during the progression of GBC [22]. In the present cohort, hepatic invasion was more frequently observed in patients exhibiting co-high expression of PD1 and TIM3 (26/57, 45.61%) (Fig. 4A). We aimed to investigate the influence of PD1 and TIM3 expression patterns on immune cell infiltration at the hepatic invasion margin (HIM) of GBC. HIM was defined as the 500 μm wide area on both sides of the invasive border (Additional file 1: Figure S6), based on previous studies [19, 20]. The density of immune cell infiltration at HIM was compared to that within the tumor core (TC) (Fig. 4B). Our results demonstrated that the group with co-high expression of PD1 and TIM3 exhibited a higher frequency of immune cell infiltration at HIM compared to the TC region (Fig. 4C). Notably, among the different expression patterns, CD8 + TILs showed the most notable preferential accumulation at HIM in cases with co-high expression of PD1 and TIM3, while no significant differences were observed in the other expression patterns. We performed multiplex immunohistochemical staining and confirmed a significantly higher density of CD8 + TIL infiltration at HIM than in TC region (Fig. 4D). Taken together, these results suggested PD1 and TIM3 co-high expression in GBC may promote the infiltration of immune cell, especially CD8 + TIL around the HIM region.

### Comparison of immune status between primary tumor and paired liver metastases

**Fig. 5:**
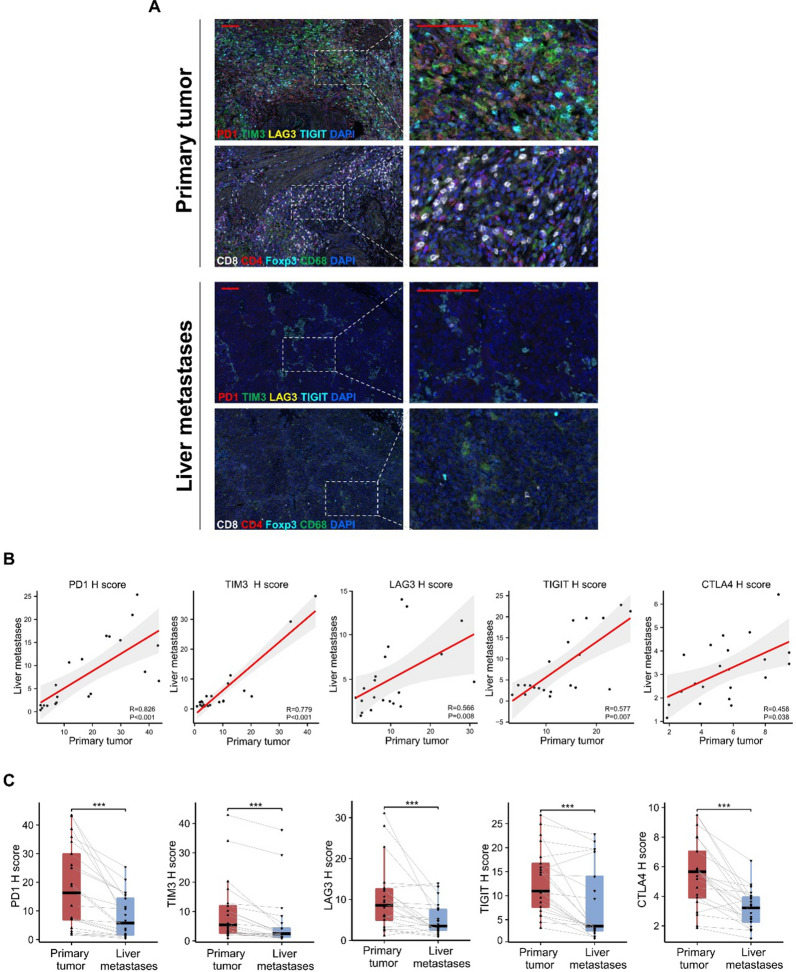
Distinct immune microenvironments in primary tumor and liver metastasis. **A**, Representative mIHC images of immune checkpoints (PD1, TIM3, TIGIT, LAG3) and Immune cell marker (CD8, CD4, CD68, Foxp3) staining within the primary tumor and its paired liver metastases (Red scale bar is 100 μm). **B**, Comparison of immune checkpoints H-score between primary tumor and liver metastasis in GBC (***P < 0.001). **C**, The correlation between the H-score of immune checkpoints in the primary tumor and that in the liver metastasis

Liver metastasis represents the most frequent form of distant metastasis in GBC, and patients with liver metastasis generally have a significantly worse prognosis [1]. Therefore, our study aimed to investigate the immune microenvironment of liver metastases in GBC. Results obtained from single and multiplex immunohistochemical staining revealed significantly lower levels of immune cell infiltration and expression of PD1, TIM3, TIGIT, LAG3, and CTLA4 in liver metastases compared to the primary tumor (Additional file 1: Figures S7, S8, and Fig. 5A). We compared the H-score of the five immune checkpoints in liver metastases and its paired primary tumor, and found that there existed a significantly positive correlation of the five immune checkpoint expressions between the liver metastases and its paired primary tumor (PD1, R = 0.826, *P* < 0.001; TIM3, R = 0.779, *P* < 0.001; TIGIT, R = 0.577, *P* = 0.007; LAG3, R = 0.779, *P* < 0.001; CTLA4, R = 0.458, *P* = 0.038) **(**Fig. 5B**)**, but the expressions were significantly lower in the liver metastases **(**Fig. 5C**)**. Furthermore, the infiltration density of CD8^+^ TIL, CD4^+^ TIL, and Foxp3^+^ TIL in liver metastases was also significantly decreased compared to the primary tumor (Additional file 1: Figure S9). Conversely, Conversely, there was a slight increase in macrophage infiltration in liver metastases, although the difference was not statistically significant (Additional file 1: Figure S9). Collectively, our findings demonstrate that both immune checkpoint expression and immune cell infiltration are markedly reduced in liver metastases compared to primary tumors in GBC.

## Discussion

In recent years, ICIs that block PD1/PDL1 have emerged as the cornerstone of comprehensive treatment for specific cancer types [23]. However, the clinical benefit of ICIs is limited to a specific group of patients with malignancies [24]. Hence, it has become increasingly critical to attain a comprehensive understanding of the immune microenvironment and explore novel combination therapeutic strategies [25]. While prior studies about GBC [26–28] have investigated the influence of immune cell infiltration and PD1/PDL1 expression on patient prognosis, there is a noticeable gap in the literature regarding the expression profiles of novel immune checkpoints in GBC tissues and their potential impact on immune microenvironment heterogeneity. Additionally, researchers are currently exploring novel immune checkpoints such as TIM3, LAG3, and TIGIT to further improve the response and utilization of ICI therapies [5], but the expression of these proteins in GBC remains largely unknown.

In this study, we conducted a comprehensive analysis of the expression of five immune checkpoints within the immune microenvironment of GBC. Our findings revealed a strong correlation between PD1 and TIM3 expression. Further analysis showed that the co-high expression of PD1 and TlM3 stands as an independent risk factor for postoperative OS. Previous studies have reported that PD1 and TlM3 can co-express on immune cells in the tumor microenvironment, defining a subset of highly inhibitory immune cells [29–31]. This phenomenon is believed to stem from a complex crosstalk mechanism among immune checkpoints within the tumor microenvironment [32]. One study tries to explain the mechanism that PD1 can sustain the presence of PD1 + TIM3 + T cells through its competitive binding with Gal9 (TIM3 ligand). This interaction leads to an increased co-expression of PD1 and TIM3 within the tumor, reshaping the immunosuppressive microenvironment [33]. The study provides clue to the mechanism of increased co-expression of PD1 and TIM3 in our study cohort.

In line with the previous study [34], we observed a positive correlation between higher levels of CD8 + TILs and improved prognosis in patients. Interestingly, our results also indicated that co-high expression of PD1 and TIM3 often correlated with increased CD8 + TIL infiltration, which initially seems contradictory. However, upon subgroup analysis, we found that within the high CD8 + TIL infiltration group, the combined expression of PD1 and TIM3 could effectively stratify patients' prognoses. Notably, this predictive ability was not statistically significant in the low CD8 + TIL infiltration group.

The highly frequent infiltration of PD1 + TIM3 + FOXP3 + cells in GBC of our cohort may explain these results. Treg cells belong to the category of immunosuppressive cells and are crucial for maintaining self-tolerance and immune homeostasis [35]. However, within the tumor immune microenvironment, continuous exposure to antigens activates Treg cells, leading to the suppression of the body's anti-tumor response and facilitating the progression of various cancer types [35]. It has been reported that PD1 is able to label dysfunctional Tregs in malignant gliomas and that PD1^hi^Tregs upregulate TIM3 expression [36]. A study on head and neck cancer [37] revealed that TIM3 + PD1 + Treg cells exhibited a heightened ability to inhibit T-cell proliferation compared to TIM3- PD1 + Treg cells, despite the high PD1 expression in both subsets. Additionally, activated Treg cells can produce cytokines such as IL-35 and IL-27, which drive the expression of PD1 and TIM3, thereby creating a profoundly suppressive tumor immune microenvironment [38–40]. It is evident that PD1 and TIM3 serve as markers of activated Treg cells [41], and the co-expression of PD1 and TIM3 enhances their immunosuppressive activity.

Regarding CD8 + TILs, activated Treg cells can influence the exhausted state of CD8 + TILs through various crosstalk mechanisms [42], either directly or indirectly. Treg cells can promote CD8 + TIL exhaustion through secreting IL-10 and IL-35 [43]. On the other hand, CXCR3 expression in Treg cells facilitates interactions with dendritic cells, leading to immunosuppression of CD8 + TILs [44]. Notably, elevated PD1 expression on Treg cells intensifies immunosuppression of CD8 + T cells by interacting with PD-L1 on CD8 + T cells [45, 46]. Therefore, we hypothesize that PD1 + TIM3 + Treg cells may contribute to the highly suppressive immune microenvironment in GBC through directly or indirectly affecting CD8 + TILs. When both PD1 and TIM3 are effectively blocked, the exhausted immune cells in the GBC microenvironment can regain their anti-tumor activity, leading to relatively prolonged OS for GBC patients.

After investigating the quantitative heterogeneity of immune components in GBC, we aimed to explore the presence of spatial heterogeneity in GBC. Our results demonstrated the existence of spatial heterogeneity of immune cells in GBC. Specifically, the accumulation of Foxp3 + TILs, CD4 + TILs, and CD68 + cells was more frequent at the hepatic invasion margin of GBC, particularly when PD1 and TIM3 were highly co-expressed. Notably, CD8 + TILs showed increased aggregation specifically at the hepatic invasion margin only when PD1 and TIM3 were highly co-expressed. While unreported in GBC studies before, the invasive margin has been established as a critical region for tumor invasion [47, 48]. Previous studies [49, 50] have reported significant immune cell distribution differences at the tumor-invasive margin, and this spatial heterogeneity have resulted in markedly different outcomes in terms of systemic therapy efficacy for patients. Our findings suggest that targeting PD1 and TIM3 may have the potential to limit liver invasion in GBC by regulating the distribution of immune cells.

The intricate crosstalk mechanisms among tumor cells, normal cells, and immune cells at the invasive margins affect the progression of the tumor [51]. Actually, the molecular and cellular mechanisms of tumor invasion in this region have not been well studied. A recent study on liver cancer [52], utilizing nanoscale-resolution Spatial Enhanced REsolution Omics-sequencing (Stereo-seq), confirmed intimate crosstalk between tumor cells at the invasive margin and neighboring normal liver cells, reshaping the local immunosuppressive microenvironment and ultimately driving tumor progression. This study enhances our understanding of tumor biology at the invasive margin, and we believe that future researchers can leverage this technology to provide valuable insights into the crosstalk mechanisms in line with the findings of our study.

Liver metastasis represents an exceedingly unfavorable prognostic factor in advanced GBC [53]. In the present study, we observed a significantly lower infiltration density of various immune cells in liver metastases compared to primary tumors. This finding aligns with previous investigations conducted in breast cancer [54] and colorectal cancer [55], suggesting that the majority of liver metastases exhibit an immunologically ‘‘cold’’ tumor phenotype. The scarcity of immune cell infiltration contributes to a diminished immune response and subsequently impairs the effectiveness of ICIs. Given the prevalent immune-desert state, the key to improving the response rate of ICIs in GBC liver metastases lies in strategies to transform ‘‘cold’’ tumors into ‘‘hot’’ tumors, thereby enabling a larger proportion of advanced GBC patients to benefit from immunotherapy, such as simultaneous blockade of PD1 and TIM3. Nevertheless, until an effective combination immunotherapy regimen is found, approaches such as chemotherapy [56] or surgical resection [57], may prove more effective than ICIs in treating liver metastases.

The present study has several limitations. Firstly, despite providing comprehensive histological evidence, there is a need for full validation of these results and exploration of the underlying molecular mechanisms in the future, utilizing techniques like CyTOF, and Stereo-seq, as well as cellular and animal experiments. Secondly, while we made efforts to minimize human bias through automated computerized scoring and other methods, future multicenter clinical studies are necessary to establish standardized norms for histological scoring. This standardization would greatly enhance the clinical utility of these biomarkers. Lastly, the sample size of liver metastases was relatively small, posing challenges for conducting more comprehensive statistical analyses.

## Conclusions

**Fig. 6:**
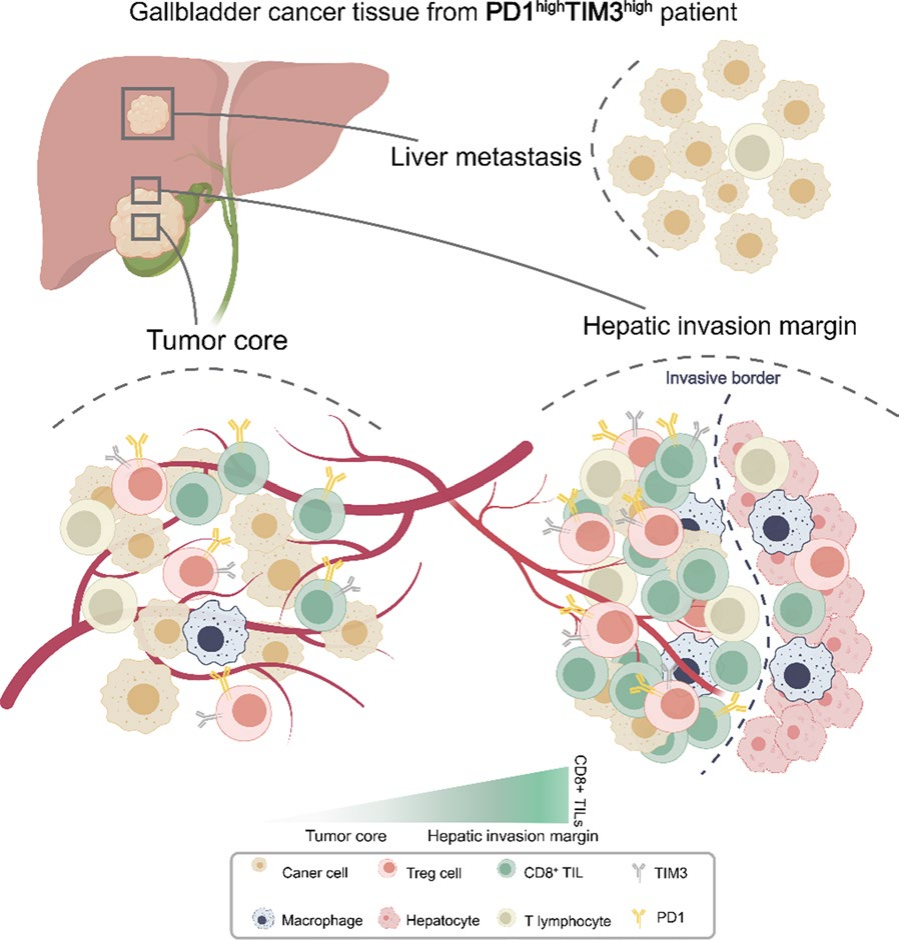
A model of tumor microenvironment heterogeneity in GBC patients with the co-high expression of PD1 and TIM3

In conclusion, our findings provide valuable evidence for the prognostic significance of PD1 and TIM3 in patients with GBC. Notably, we observed extensive colocalization of PD1 and TIM3 on Foxp3 + TILs within GBC tissue. This colocalization may play a role in modifying the spatial and quantitative heterogeneity of CD8 + TILs and Foxp3 + TILs in the GBC microenvironment. Additionally, our study reveals the immunologically "cold" status in liver metastases of GBC **(**Fig. 6**)**. These findings enhance our comprehension of the immune microenvironment in GBC and offer insights into potential novel therapeutic targets for this disease.

### Supplementary Information

**Supplementary Materials Table S1.** Information on the primary antibodies used in IHC and their incubation message. **Table S2.** Optimal cut-off value for the ICP H-score and immune cell density obtained by *X-TILE* software. **Table S3.** Clinicopathological characteristics of GBC study cohort. **Table S4.** The correlation between CTLA4, LAG3, TIGIT expression status and clinicopathological characteristics in GBC. **Figure S1.** Kaplan-Meier survival curves for postoperative OS of GBC patients according to TIGIT, CTLA4, and LAG3 expression status. **Figure S2.** The chord diagram shows the correlation network of immune cell density in GBC tissue. The band represents a positive correlation between the ICP and immune cell density, and the width indicates the magnitude of the Pearson’s correlation coefficient (the *P* value for testing the correlation coefficient was < 0.05). **Figure S3.** The correlation of PD1 and TIM3 expression status and CD8+TIL, CD4+TIL, CD68+cell, Foxp3+TIL density in GBC tissue (*P < 0.05; **P < 0.01; ***P < 0.001). **Figure S4.** The percentage of CD8+TIL with different PD1/TIM3 co-expression status in GBC tissues. **Figure S5.** Kaplan-Meier survival curves for postoperative OS of GBC patients according to CD8+TIL, CD4+TIL, CD68+ cell, Foxp3+TIL infiltration density. **Figure S6.** Panoramic scan of the section of a tumor with liver invasion. The white dotted line marks the line of liver invasion and the red area of 1000μm width is defined as the Hepatic invasion margin. **Figure S7.** Representative IHC images of PD1, TIM3, TIGIT, LAG3, CTLA4 staining are shown from the same GBC patient who had primary tumor (left column) and liver metastases (right column). **Figure S8.** Representative IHC images of CD8, CD4, CD68, Foxp3 staining are shown from the same GBC patient who had primary tumor (left column) and liver metastases (right column). **Figure S9.** Comparison of Immune cell counts between primary tumor and liver metastases in GBC (***P < 0.001; ns: no significance).

**Image Analysis Protocol **Detailed method for QuPath image analysis of immune. Detailed method for QuPath image analysis of immune. Detailed method for INFO image analysis of distinguishing.

## Data Availability

The data can be obtained from the corresponding author on a reasonable request.

## Abbreviations

ORR: Object response rate
GBC: Gallbladder cancer
ICI: Immune checkpoint inhibitor
OS: Overall survival
IHC: Immunohistochemistry
mIHC: Multiplex immunohistochemistry
K-M: Kaplan Meier
HIM: Hepatic invasion margin
TC: Tumor core
PD1: Programmed death 1
TIM3: T cell immunoglobulin domain and mucin domain 3
LAG3: Lymphocyte activation gene 3
TIGIT: T cell immunoreceptor with Ig and ITIM domains
CTLA4: Cytotoxic T lymphocyte antigen 4
ICP: Immune checkpoint protein

## References

[1] Roa JC, García P, Kapoor VK, Maithel SK, Javle M, Koshiol J (2022). Gallbladder cancer. Nature Rev Dis Primers.

[2] Valle JW, Kelley RK, Nervi B, Oh DY, Zhu AX (2021). Biliary tract cancer. Lancet.

[3] Zhang W, Zhou H, Wang Y, Zhang Z, Cao G, Song T (2020). Systemic treatment of advanced or recurrent biliary tract cancer. Biosci Trends.

[4] Vivekanandhan S, Bahr D, Kothari A, Ashary MA, Baksh M, Gabriel E (2023). Immunotherapies in rare cancers. Mol Cancer.

[5] Kraehenbuehl L, Weng CH, Eghbali S, Wolchok JD, Merghoub T (2022). Enhancing immunotherapy in cancer by targeting emerging immunomodulatory pathways. Nat Rev Clin Oncol.

[6] Curigliano G, Gelderblom H, Mach N, Doi T, Tai WMD, Forde P (2019). Abstract CT183: Phase (Ph) I/II study of MBG453± spartalizumab (PDR001) in patients (pts) with advanced malignancies. Cancer Res.

[7] Falchook GS, Ribas A, Davar D, Eroglu Z, Wang JS, Luke JJ (2022). Phase 1 trial of TIM-3 inhibitor cobolimab monotherapy and in combination with PD-1 inhibitors nivolumab or dostarlimab (AMBER). J Clin Oncol.

[8] Niu J, Maurice-Dror C, Lee DH, Kim DW, Nagrial A, Voskoboynik M (2022). First-in-human phase 1 study of the anti-TIGIT antibody vibostolimab as monotherapy or with pembrolizumab for advanced solid tumors, including non-small-cell lung cancer(☆). Ann Oncol Off J Eur Soc Med Oncol.

[9] Felip E, Majem M, Doger B, Clay TD, Carcereny E, Bondarenko I (2022). A phase II study (TACTI-002) in first-line metastatic non–small cell lung carcinoma investigating eftilagimod alpha (soluble LAG-3 protein) and pembrolizumab: updated results from a PD-L1 unselected population. J Clin Oncol.

[10] Zhou Q, Munger ME, Veenstra RG, Weigel BJ, Hirashima M, Munn DH (2011). Coexpression of Tim-3 and PD-1 identifies a CD8+ T-cell exhaustion phenotype in mice with disseminated acute myelogenous leukemia. Blood.

[11] Sun F, Guo ZS, Gregory AD, Shapiro SD, Xiao G, Qu Z (2020). Dual but not single PD-1 or TIM-3 blockade enhances oncolytic virotherapy in refractory lung cancer. J Immunother Cancer.

[12] Yiong CS, Lin TP, Lim VY, Toh TB, Yang VS (2023). Biomarkers for immune checkpoint inhibition in sarcomas - are we close to clinical implementation?. Biomarker research.

[13] Doroshow DB, Bhalla S, Beasley MB, Sholl LM, Kerr KM, Gnjatic S (2021). PD-L1 as a biomarker of response to immune-checkpoint inhibitors. Nat Rev Clin Oncol.

[14] Teng MW, Ngiow SF, Ribas A, Smyth MJ (2015). Classifying cancers based on T-cell infiltration and PD-L1. Can Res.

[15] Topalian SL, Forde PM, Emens LA, Yarchoan M, Smith KN, Pardoll DM (2023). Neoadjuvant immune checkpoint blockade: a window of opportunity to advance cancer immunotherapy. Cancer Cell.

[16] Cheng H, Janakiram M, Borczuk A, Lin J, Qiu W, Liu H (2017). HHLA2, a new immune checkpoint member of the B7 family, is widely expressed in human lung cancer and associated with egfr mutational status. Clin Cancer Res Off J Am Assoc Cancer Res.

[17] Bankhead P, Loughrey MB, Fernández JA, Dombrowski Y, McArt DG, Dunne PD (2017). QuPath: open source software for digital pathology image analysis. Sci Rep.

[18] Parra ER, Behrens C, Rodriguez-Canales J, Lin H, Mino B, Blando J (2016). Image analysis-based assessment of PD-L1 and tumor-associated immune cells density supports distinct intratumoral microenvironment groups in non-small cell lung carcinoma patients. Clin Cancer Res Off J Am Assoc Cancer Res.

[19] Nearchou IP, Gwyther BM, Georgiakakis ECT, Gavriel CG, Lillard K, Kajiwara Y (2020). Spatial immune profiling of the colorectal tumor microenvironment predicts good outcome in stage II patients. NPJ Digital Med.

[20] Wu L, Yan J, Bai Y, Chen F, Zou X, Xu J (2023). An invasive zone in human liver cancer identified by Stereo-seq promotes hepatocyte-tumor cell crosstalk, local immunosuppression and tumor progression. Cell Res.

[21] Camp RL, Dolled-Filhart M, Rimm DL (2004). X-tile: a new bio-informatics tool for biomarker assessment and outcome-based cut-point optimization. Clin Cancer Res Off J Am Assoc Cancer Res.

[22] Zhou Y, Yuan K, Yang Y, Ji Z, Zhou D, Ouyang J (2023). Gallbladder cancer: current and future treatment options. Front Pharmacol.

[23] Pinter M, Scheiner B, Pinato DJ (2023). Immune checkpoint inhibitors in hepatocellular carcinoma: emerging challenges in clinical practice. Lancet Gastroenterol Hepatol.

[24] Kim TK, Vandsemb EN, Herbst RS, Chen L (2022). Adaptive immune resistance at the tumour site: mechanisms and therapeutic opportunities. Nat Rev Drug Discovery.

[25] Galon J, Bruni D (2019). Approaches to treat immune hot, altered and cold tumours with combination immunotherapies. Nat Rev Drug Discovery.

[26] Fluxá P, Rojas-Sepúlveda D, Gleisner MA, Tittarelli A, Villegas P, Tapia L (2018). High CD8(+) and absence of Foxp3(+) T lymphocytes infiltration in gallbladder tumors correlate with prolonged patients survival. BMC Cancer.

[27] Neyaz A, Husain N, Kumari S, Gupta S, Shukla S, Arshad S (2018). Clinical relevance of PD-L1 expression in gallbladder cancer: a potential target for therapy. Histopathology.

[28] Bo X, Wang J, Wang C, Nan L, Gao Z, Xin Y (2020). High infiltration of mast cells is associated with improved response to adjuvant chemotherapy in gallbladder cancer. Cancer Sci.

[29] Afanasiev OK, Yelistratova L, Miller N, Nagase K, Paulson K, Iyer JG (2013). Merkel polyomavirus-specific T cells fluctuate with merkel cell carcinoma burden and express therapeutically targetable PD-1 and Tim-3 exhaustion markers. Clinical cancer Res Off J Am Assoc Cancer Res.

[30] Sakuishi K, Apetoh L, Sullivan JM, Blazar BR, Kuchroo VK, Anderson AC (2010). Targeting Tim-3 and PD-1 pathways to reverse T cell exhaustion and restore anti-tumor immunity. J Exp Med.

[31] Fourcade J, Sun Z, Benallaoua M, Guillaume P, Luescher IF, Sander C (2010). Upregulation of Tim-3 and PD-1 expression is associated with tumor antigen-specific CD8+ T cell dysfunction in melanoma patients. J Exp Med.

[32] Gaikwad S, Agrawal MY, Kaushik I, Ramachandran S, Srivastava SK (2022). Immune checkpoint proteins: signaling mechanisms and molecular interactions in cancer immunotherapy. Semin Cancer Biol.

[33] Yang R, Sun L, Li CF, Wang YH, Yao J, Li H (2021). Galectin-9 interacts with PD-1 and TIM-3 to regulate T cell death and is a target for cancer immunotherapy. Nat Commun.

[34] Lin J, Long J, Wan X, Chen J, Bai Y, Wang A (2018). Classification of gallbladder cancer by assessment of CD8(+) TIL and PD-L1 expression. BMC Cancer.

[35] Kang JH, Zappasodi R (2023). Modulating Treg stability to improve cancer immunotherapy. Trends Cancer.

[36] Lowther DE, Goods BA, Lucca LE, Lerner BA, Raddassi K, van Dijk D (2016). PD-1 marks dysfunctional regulatory T cells in malignant gliomas. JCI Insight.

[37] Liu Z, McMichael EL, Shayan G, Li J, Chen K, Srivastava R (2018). Novel effector phenotype of Tim-3(+) regulatory T cells leads to enhanced suppressive function in head and neck cancer patients. Clin Cancer Res Off J Am Assoc Cancer Res.

[38] Turnis ME, Sawant DV, Szymczak-Workman AL, Andrews LP, Delgoffe GM, Yano H (2016). Interleukin-35 limits anti-tumor immunity. Immunity.

[39] Zhu C, Sakuishi K, Xiao S, Sun Z, Zaghouani S, Gu G (2015). An IL-27/NFIL3 signalling axis drives Tim-3 and IL-10 expression and T-cell dysfunction. Nat Commun.

[40] Chihara N, Madi A, Kondo T, Zhang H, Acharya N, Singer M (2018). Induction and transcriptional regulation of the co-inhibitory gene module in T cells. Nature.

[41] Gupta S, Thornley TB, Gao W, Larocca R, Turka LA, Kuchroo VK (2012). Allograft rejection is restrained by short-lived TIM-3+PD-1+Foxp3+ Tregs. J Clin Investig.

[42] Li C, Jiang P, Wei S, Xu X, Wang J (2020). Regulatory T cells in tumor microenvironment: new mechanisms, potential therapeutic strategies and future prospects. Mol Cancer.

[43] Sawant DV, Yano H, Chikina M, Zhang Q, Liao M, Liu C (2019). Adaptive plasticity of IL-10(+) and IL-35(+) T(reg) cells cooperatively promotes tumor T cell exhaustion. Nat Immunol.

[44] Moreno Ayala MA, Campbell TF, Zhang C, Dahan N, Bockman A, Prakash V (2023). CXCR3 expression in regulatory T cells drives interactions with type I dendritic cells in tumors to restrict CD8(+) T cell antitumor immunity. Immunity.

[45] Park HJ, Park JS, Jeong YH, Son J, Ban YH, Lee BH (2015). PD-1 upregulated on regulatory T cells during chronic virus infection enhances the suppression of CD8+ T cell immune response via the interaction with PD-L1 expressed on CD8+ T cells. J Immunol.

[46] Kim HR, Park HJ, Son J, Lee JG, Chung KY, Cho NH (2019). Tumor microenvironment dictates regulatory T cell phenotype: upregulated immune checkpoints reinforce suppressive function. J Immunother Cancer.

[47] Laghi L, Bianchi P, Miranda E, Balladore E, Pacetti V, Grizzi F (2009). CD3+ cells at the invasive margin of deeply invading (pT3-T4) colorectal cancer and risk of post-surgical metastasis: a longitudinal study. Lancet Oncol.

[48] Schürch CM, Bhate SS, Barlow GL, Phillips DJ, Noti L, Zlobec I (2020). Coordinated cellular neighborhoods orchestrate antitumoral immunity at the colorectal cancer invasive front. Cell.

[49] Nearchou IP, Lillard K, Gavriel CG, Ueno H, Harrison DJ, Caie PD (2019). Automated analysis of lymphocytic infiltration, tumor budding, and their spatial relationship improves prognostic accuracy in colorectal cancer. Cancer Immunol Res.

[50] Halama N, Michel S, Kloor M, Zoernig I, Benner A, Spille A (2011). Localization and density of immune cells in the invasive margin of human colorectal cancer liver metastases are prognostic for response to chemotherapy. Can Res.

[51] Quail DF, Joyce JA (2013). Microenvironmental regulation of tumor progression and metastasis. Nat Med.

[52] Wu L, Yan J, Bai Y, Chen F, Zou X, Xu J (2023). An invasive zone in human liver cancer identified by stereo-seq promotes hepatocyte-tumor cell crosstalk, local immunosuppression and tumor progression. Cell Res.

[53] Harding JJ, Khalil DN, Fabris L, Abou-Alfa GK (2023). Rational development of combination therapies for biliary tract cancers. J Hepatol.

[54] Ogiya R, Niikura N, Kumaki N, Bianchini G, Kitano S, Iwamoto T (2016). Comparison of tumor-infiltrating lymphocytes between primary and metastatic tumors in breast cancer patients. Cancer Sci.

[55] Shibutani M, Maeda K, Nagahara H, Fukuoka T, Matsutani S, Kashiwagi S (2018). A comparison of the local immune status between the primary and metastatic tumor in colorectal cancer: a retrospective study. BMC Cancer.

[56] You MS, Ryu JK, Choi YH, Choi JH, Huh G, Paik WH (2019). Therapeutic outcomes and prognostic factors in unresectable gallbladder cancer treated with gemcitabine plus cisplatin. BMC Cancer.

[57] Higuchi R, Ono H, Matsuyama R, Takemura Y, Kobayashi S, Otsubo T (2022). Examination of the characteristics of long-term survivors among patients with gallbladder cancer with liver metastasis who underwent surgical treatment: a retrospective multicenter study (ACRoS1406). BMC Gastroenterol.

