# Correction to: Increased co-expression of PD1 and TIM3 is associated with poor prognosis and immune microenvironment heterogeneity in gallbladder cancer

**DOI:** 10.1186/s12967-024-04905-5

**Published:** 2024-01-30

**Authors:** Xing He, Yaorong Peng, Gui He, Huilin Ye, Liqiang Liu, Qixian Zhou, Juanyi Shi, Sha Fu, Jie Wang, Zhenyu Zhou, Wenbin Li

**Affiliations:** 1 Department of Biliary and Pancreatic Surgery, Sun Yat-sen Memorial Hospital, Sun Yat-sen University, No.107 Yanjiang West Road, Yuexiu District, Guangzhou, 510120 Guangdong People’s Republic of China; 2 Department of Hepatobiliary Surgery, Sun Yat-sen Memorial Hospital, Sun Yat-sen University, Guangzhou, 510120 People’s Republic of China; 3 Guangdong Provincial Key Laboratory of Malignant Tumor Epigenetics and Gene Regulation, Sun Yat-sen Memorial Hospital, Sun Yat-sen University, Guangzhou, 510120 People’s Republic of China; 4 Cellular & Molecular Diagnostics Center, Sun Yat-sen Memorial Hospital, Sun Yat-sen University, Guangzhou, 510120 People’s Republic of China

**Correction: Journal of Translational Medicine (2023) 21:717** 10.1186/s12967-023-04589-3

Following publication of the original article [1], we have been notified that the article was published without the sentence of authors’ contribution.

It should be as follows:

^†^Xing He, Yaorong Peng, and Gui He contributed equally to this work.

The original article [1] was updated.
