# Supplementary material for: Increased co-expression of PD1 and TIM3 is associated with poor prognosis and immune microenvironment heterogeneity in gallbladder cancer
Source: J Transl Med. 2023 Oct 12;21:717. doi: 10.1186/s12967-023-04589-3 (PMC10571407; doi:10.1186/s12967-023-04589-3)
Supplement: Supplementary file 1 — Supplementary Materials Table S1. Information on the primary antibodies used in IHC and their incubation message. Table S2. Optimal cut-off value for the ICP H-score and immune cell density obtained by X-TILE software. Table S3. Clinicopathological characteristics of GBC study cohort. Table S4. The correlation between CTLA4, LAG3, TIGIT expression status and clinicopathological characteristics in GBC. Figure S1. Kaplan-Meier survival curves for postoperative OS of GBC patients according to TIGIT, CTLA4, and LAG3 expression status. Figure S2. The chord diagram shows the correlation network of immune cell density in GBC tissue. The band represents a positive correlation between the ICP and immune cell density, and the width indicates the magnitude of the Pearson’s correlation coefficient (the P value for testing the correlation coefficient was < 0.05). Figure S3. The correlation of PD1 and TIM3 expression status and CD8+TIL, CD4+TIL, CD68+cell, Foxp3+TIL density in GBC tissue (*P < 0.05; **P < 0.01; ***P < 0.001). Figure S4. The percentage of CD8+TIL with different PD1/TIM3 co-expression status in GBC tissues. Figure S5. Kaplan-Meier survival curves for postoperative OS of GBC patients according to CD8+TIL, CD4+TIL, CD68+ cell, Foxp3+TIL infiltration density. Figure S6. Panoramic scan of the section of a tumor with liver invasion. The white dotted line marks the line of liver invasion and the red area of 1000μm width is defined as the Hepatic invasion margin. Figure S7. Representative IHC images of PD1, TIM3, TIGIT, LAG3, CTLA4 staining are shown from the same GBC patient who had primary tumor (left column) and liver metastases (right column). Figure S8. Representative IHC images of CD8, CD4, CD68, Foxp3 staining are shown from the same GBC patient who had primary tumor (left column) and liver metastases (right column). Figure S9. Comparison of Immune cell counts between primary tumor and liver metastases in GBC (***P < 0.001; ns: no significance). [file 12967_2023_4589_MOESM1_ESM.docx]

|  | | |
| --- | --- | --- |
| **Table S1. Information on the primary antibodies used in IHC and their incubation message.** | | |
| Antibody | Group | Message |
| TIM3(D5D5R™) Rabbit mAb | Cell Signaling Technology, Inc（#45208S） | 1:400  12h at 4℃ |
| PD1(D5D5R™) Mouse mAb | Proteintech Group, Inc（#66220-1-Ig） | 1:4000  12h at 4℃ |
| LAG3 Rabbit pAb | Proteintech Group, Inc（#16616-1-AP） | 1:2000  12h at 4℃ |
| CTLA4(E2V1Z) Rabbit mAb | Cell Signaling Technology, Inc（#53560S） | 1:200  12h at 4℃ |
| TIGIT(E5Y1W) Rabbit mAb | Cell Signaling Technology, Inc（#99567S） | 1:400  12h at 4℃ |
| Foxp3(236A/E7) Mouse mAb | Abcam，Inc  （#ab20034） | 1：300  12h at 4℃ |
| CD8(1G2B10) Mouse mAb | Proteintech Group, Inc  （#66868-1-IG） | 1:8000  12h at 4℃ |
| CD4(2H4A2) Mouse mAb | Proteintech Group, Inc  （#67786-1-IG） | 1:1000  12h at 4℃ |
| CD68 Rabbit pAb | Proteintech Group, Inc  （# 28058-1-AP） | 1:1000  12h at 4℃ |

| **Table S2. Optimal cut-off value for the ICP H-score and immune cell density obtained by *X-TILE* software.** | |
| --- | --- |
| Variable | Cut-off value |
| PD1 H score | 6.5 |
| TIM3 H score | 4.9 |
| LAG3 H score | 11.6 |
| CTLA4 H score | 5.6 |
| TIGIT H score | 16 |
| CD8+TIL density | 200 |
| CD4+TIL density | 179.5 |
| CD68+ cell density | 52 |
| Foxp3+TIL density | 52.5 |

| **Table S3. Clinicopathological characteristics of GBC study cohort.的临** | |  |
| --- | --- | --- |
| Characteristics | All patient [ cases (%)] |  |
|  |  |  |
| Total | 127(100.0) |  |
| Gender |  |  |
| female | 61 (48.0) |  |
| male | 66 (52.0) |  |
| Age |  |  |
| ≤ 60 years | 50 (39.4) |  |
| > 60 years | 77 (60.6) |  |
| Jaundice |  |  |
| no | 85 (66.9) |  |
| yes | 42 (33.1) |  |
| Gall stone |  |  |
| no | 66 (52.0) |  |
| yes | 61 (48.0) |  |
| Cholecystitis |  |  |
| no | 87 (68.5) |  |
| yes | 40 (31.5) |  |
| Abdominal discomfort |  |  |
| no | 46 (36.2) |  |
| yes | 81 (63.8) |  |
| Tumor size |  |  |
| ≥ 3 cm | 67 (52.8) |  |
| < 3 cm | 60 (47.2) |  |
| Tumor main location |  |  |
| body | 63 (49.6) |  |
| cystic | 10 (7.9) |  |
| fundus | 38 (29.9) |  |
| neck | 16 (12.6) |  |
| Microvascular invasion |  |  |
| no | 75 (59.1) |  |
| yes | 52 (40.9) |  |
| Perineural invasion |  |  |
| no | 67 (52.8) |  |
| yes | 60 (47.2) |  |
| Hepatic invasion |  |  |
| no | 76 (59.8) |  |
| yes | 51 (40.2) |  |
| Surgical margin |  |  |
| R0 | 88 (69.3) |  |
| R1 | 39(30.7) |  |
| T |  |  |
| 1a | 5 (3.9) |  |
| 1b | 5 (3.9) |  |
| 2a | 13 (10.2) |  |
| 2b | 23 (18.1) |  |
| 3 | 60 (47.3) |  |
| 4 | 21 (16.6) |  |
| N |  |  |
| 0 | 52 (40.9) |  |
| 1 | 31 (24.4) |  |
| 2 | 44 (34.6) |  |
| M |  |  |
| 0 | 94 (74.0) |  |
| 1 | 33 (26.0) |  |
| TNM stage |  |  |
| Ⅰ | 10 (7.9) |  |
| Ⅱ | 23 (18.1) |  |
| Ⅲ | 26 (20.5) |  |
| Ⅳ | 68 (53.5) |  |
| Histological differentiation |  |  |
| poor | 52 (40.9) |  |
| moderate | 47 (37.0) |  |
| well | 28 (22.0) |  |
| CA199 |  |  |
| negative | 54 (42.5) |  |
| positive | 73 (57.5) |  |
| CEA |  |  |
| negative | 82 (64.6) |  |
| positive | 1. (35.4) |  |
| Adjuvant therapy |  |  |
| chemotherapy | 74 (58.3) |  |
| chemotherapy&radiotherapy | 2 (1.5) |  |
| no | 51(40.2) |  |

| **Table S4. The correlation between CTLA4, LAG3, TIGIT expression status and clinicopathological characteristics in GBC.** | | | | | | | | | | | |
| --- | --- | --- | --- | --- | --- | --- | --- | --- | --- | --- | --- |
| **Characteristic** |  |  | **CTLA4 expression** | | | **LAG3 expression** | | | **TIGIT expression** | | |
|  |  | Cases.n | High | Low | P value | High | Low | P value | High | Low | P value |
| Gender | |  |  |  | 0.869 |  |  | 0.104 |  |  | 0.706 |
| Female | | 61 | 25 | 36 |  | 27 | 34 |  | 22 | 39 |  |
| Male | | 66 | 28 | 38 |  | 19 | 47 |  | 27 | 39 |  |
| Age | |  |  |  | 0.547 |  |  | 0.883 |  |  | 0.938 |
| < 60 years | | 50 | 23 | 27 |  | 19 | 31 |  | 20 | 30 |  |
| > 60 years | | 77 | 30 | 47 |  | 27 | 50 |  | 29 | 48 |  |
| Tumor size | |  |  |  | 0.212 |  |  | 0.513 |  |  | 0.622 |
| > 3 cm | | 67 | 24 | 43 |  | 22 | 45 |  | 24 | 43 |  |
| < 3 cm | | 60 | 29 | 31 |  | 24 | 36 |  | 25 | 35 |  |
| Microvascular invasion | |  |  |  | 0.164 |  |  | 0.169 |  |  | 0.981 |
| no | | 75 | 27 | 48 |  | 23 | 52 |  | 29 | 46 |  |
| yes | | 52 | 26 | 26 |  | 23 | 29 |  | 20 | 32 |  |
| Perineural invasion | |  |  |  | 0.599 |  |  | 0.164 |  |  | 0.221 |
| no | | 67 | 26 | 41 |  | 20 | 47 |  | 22 | 45 |  |
| yes | | 60 | 27 | 33 |  | 26 | 34 |  | 27 | 33 |  |
| T | |  |  |  | **0.033** |  |  | **0.018** |  |  | 0.394 |
| Low(1.2) | | 46 | 13 | 33 |  | 10 | 36 |  | 15 | 31 |  |
| High(3.4) | | 81 | 40 | 41 |  | 36 | 45 |  | 34 | 47 |  |
| N | |  |  |  | 0.124 |  |  | 0.104 |  |  | 0.342 |
| Low(0) | | 52 | 17 | 35 |  | 14 | 38 |  | 17 | 35 |  |
| High(1.2) | | 75 | 36 | 39 |  | 32 | 43 |  | 32 | 43 |  |
| M | |  |  |  | 0.126 |  |  | 0.818 |  |  | 0.179 |
| Absent(0) | | 94 | 35 | 59 |  | 33 | 61 |  | 40 | 54 |  |
| Present(1) | | 33 | 18 | 15 |  | 13 | 20 |  | 9 | 24 |  |
| TNM stage | |  |  |  | **0.027** |  |  | **0.030** |  |  | 0.923 |
| Low(Ⅰ.Ⅱ.Ⅲ) | | 59 | 18 | 41 |  | 15 | 44 |  | 22 | 37 |  |
| High(Ⅳ) | | 68 | 35 | 33 |  | 31 | 37 |  | 27 | 41 |  |
| Histologic differentiation | | |  |  | 0.079 |  |  | 0.532 |  |  | 0.366 |
| Moderate. Well | | 75 | 26 | 49 |  | 25 | 50 |  | 26 | 49 |  |
| Poor | | 52 | 27 | 25 |  | 21 | 31 |  | 23 | 29 |  |
| CA19-9 | |  |  |  | 0.475 |  |  | 0.442 |  |  | 0.623 |
| Negative | | 54 | 25 | 29 |  | 17 | 37 |  | 19 | 35 |  |
| Positive | | 73 | 28 | 45 |  | 29 | 44 |  | 30 | 43 |  |
| CEA | |  |  |  | 0.786 |  |  | 0.643 |  |  | 0.958 |
| Negative | | 82 | 33 | 49 |  | 28 | 54 |  | 31 | 51 |  |
| Positive | | 45 | 20 | 25 |  | 18 | 27 |  | 18 | 27 |  |

**Figure S1**


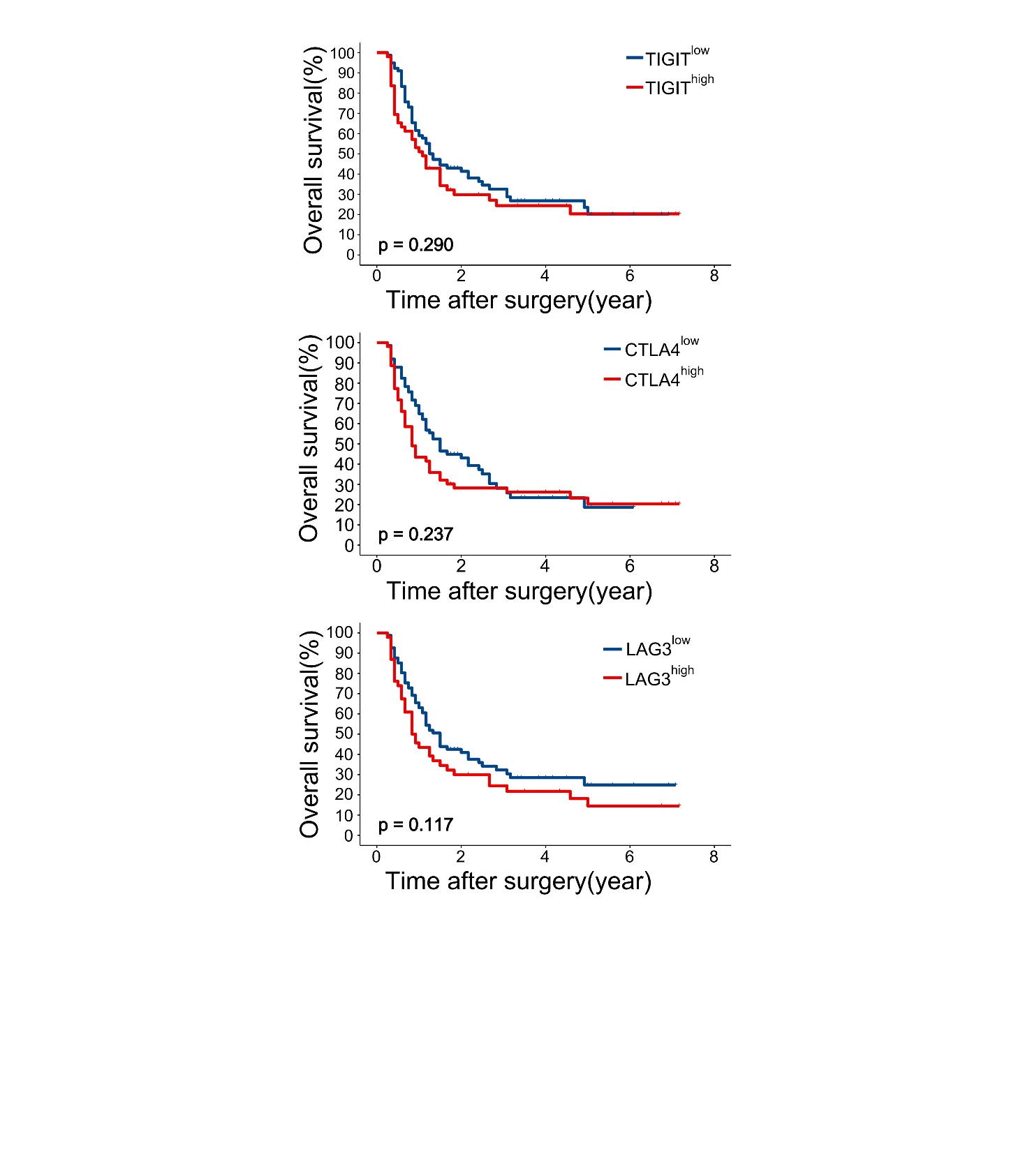


**Figure S1.**  Kaplan-Meier survival curves for postoperative OS of GBC patients according to TIGIT, CTLA4, and LAG3 expression status.

**Figure S2**


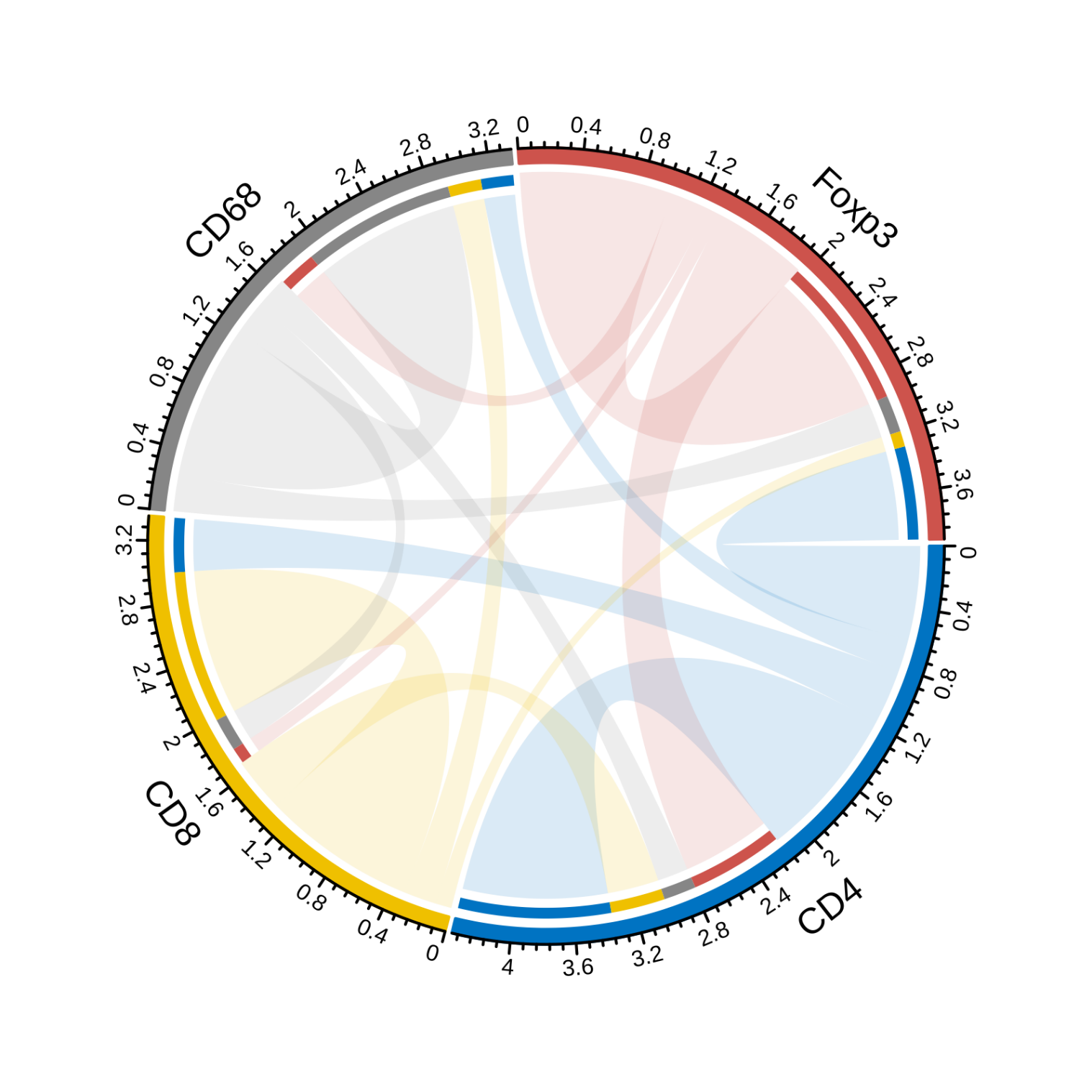


**Figure S2.**  The chord diagram shows the correlation network of immune cell density in GBC tissue. The band represents a positive correlation between the ICP and immune cell density, and the width indicates the magnitude of the Pearson’s correlation coefficient (the *P* value for testing the correlation coefficient was < 0.05).

**Figure S3**

**
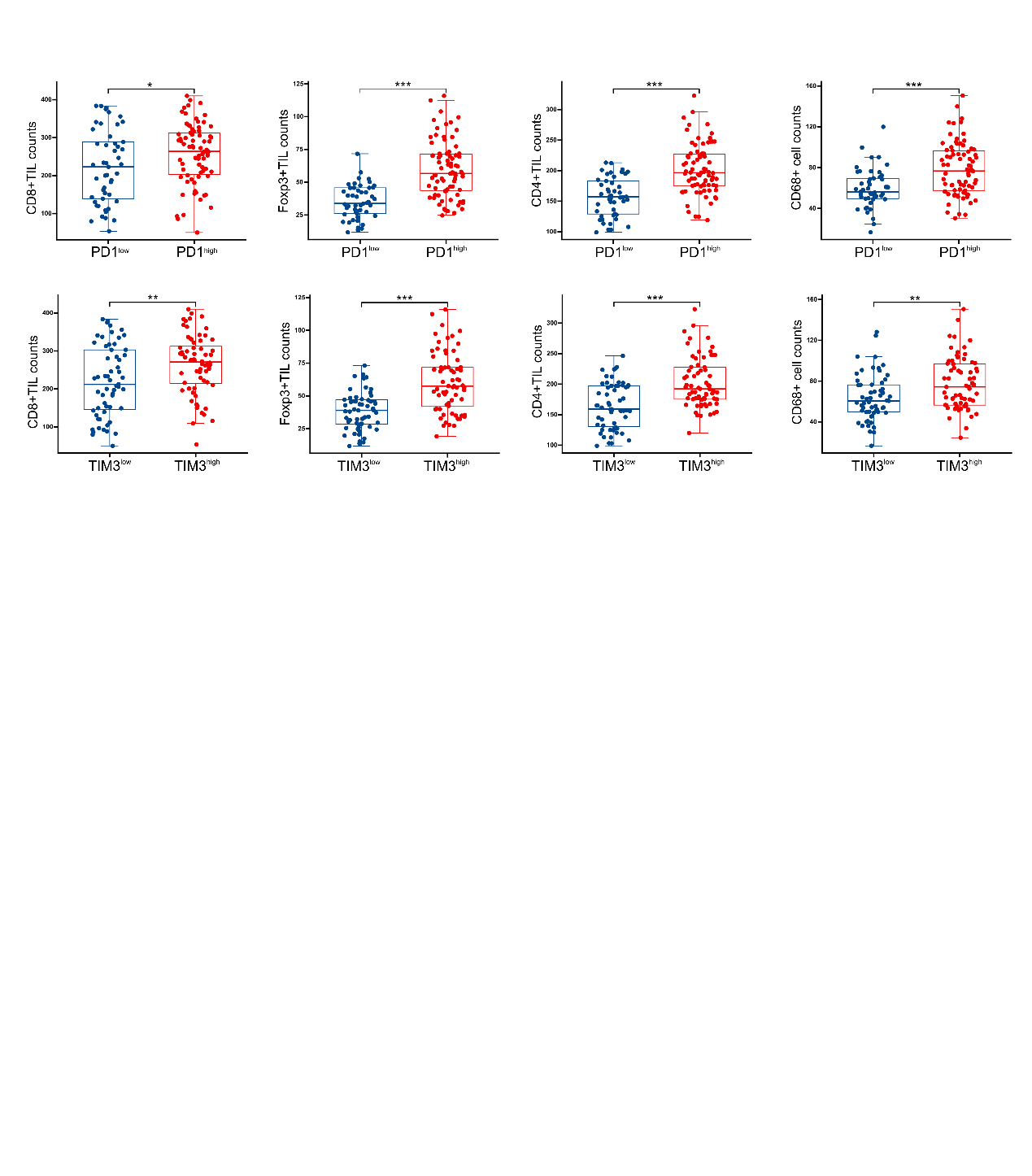
**

**Figure S3.**  The correlation of PD1 and TIM3 expression status and CD8+TIL, CD4+TIL, CD68+cell, Foxp3+TIL density in GBC tissue (**P* < 0.05; ***P* < 0.01; ****P* < 0.001).

**Figure S4**

**
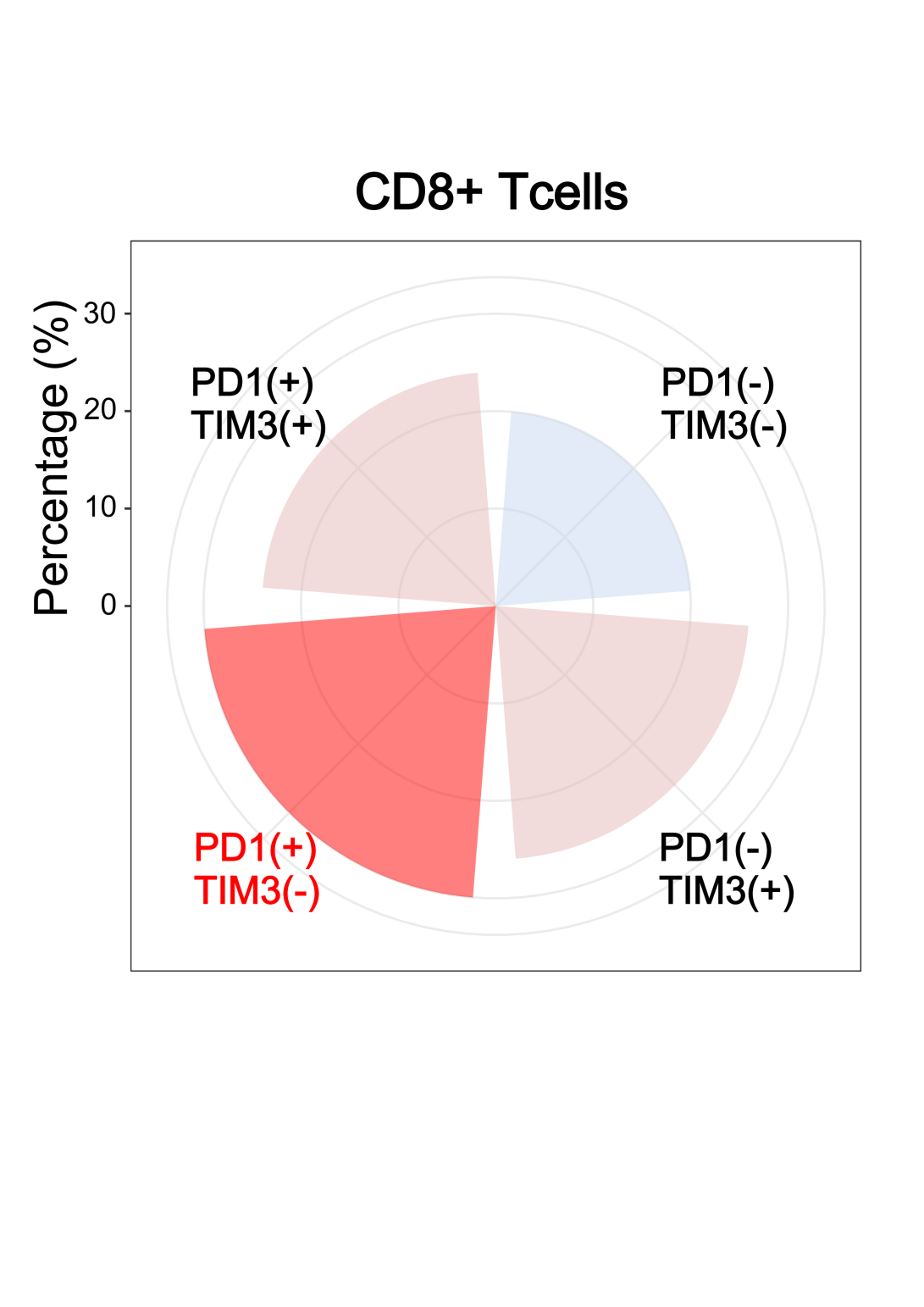
**

**Figure S4.**  The percentage of CD8+TIL with different PD1/TIM3 co-expression status in GBC tissues.

**Figure S5**


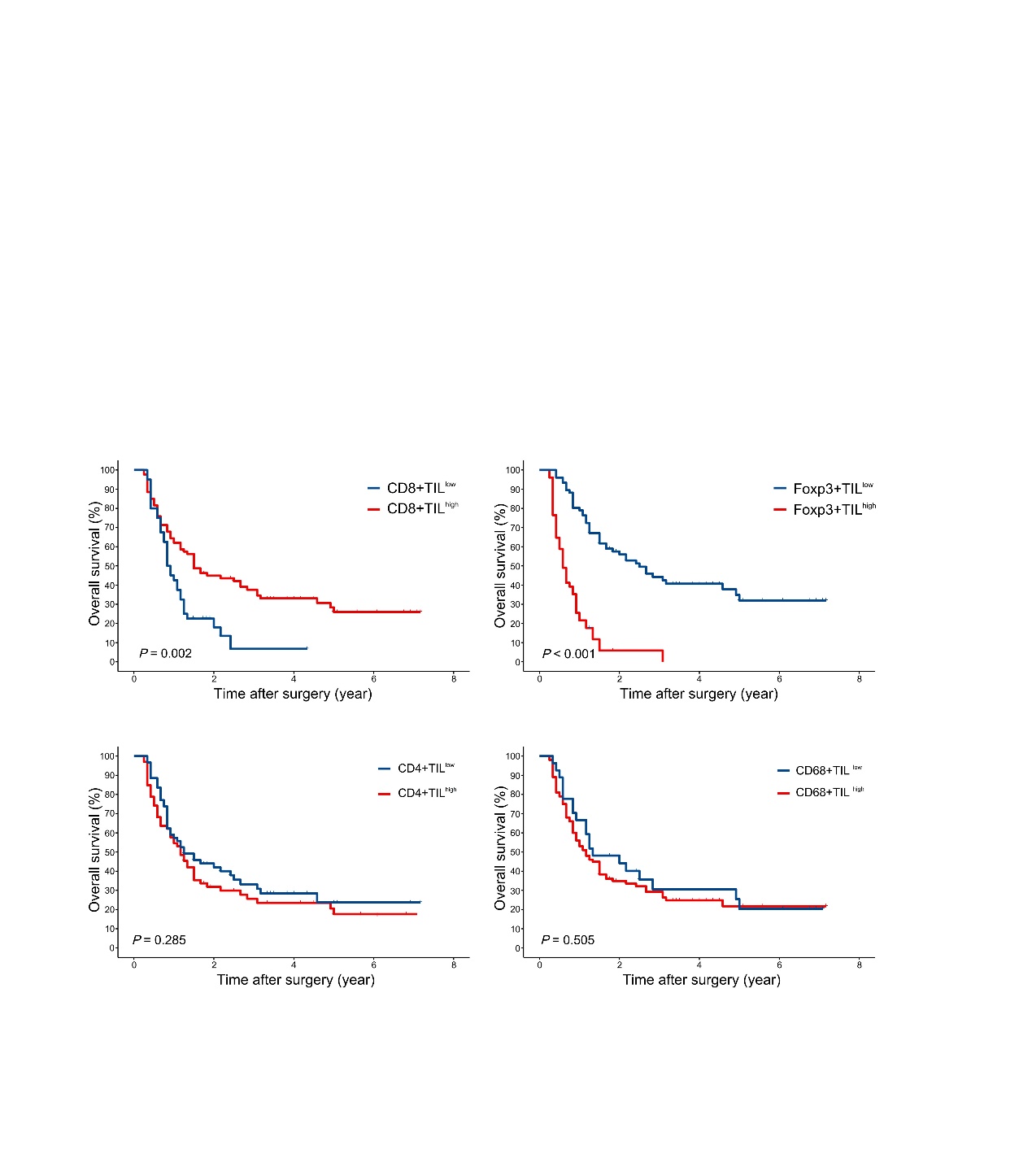


**Figure S5.** Kaplan-Meier survival curves for postoperative OS of GBC patients according to CD8+TIL, CD4+TIL, CD68+ cell, Foxp3+TIL infiltration density.

**Figure S6**

**
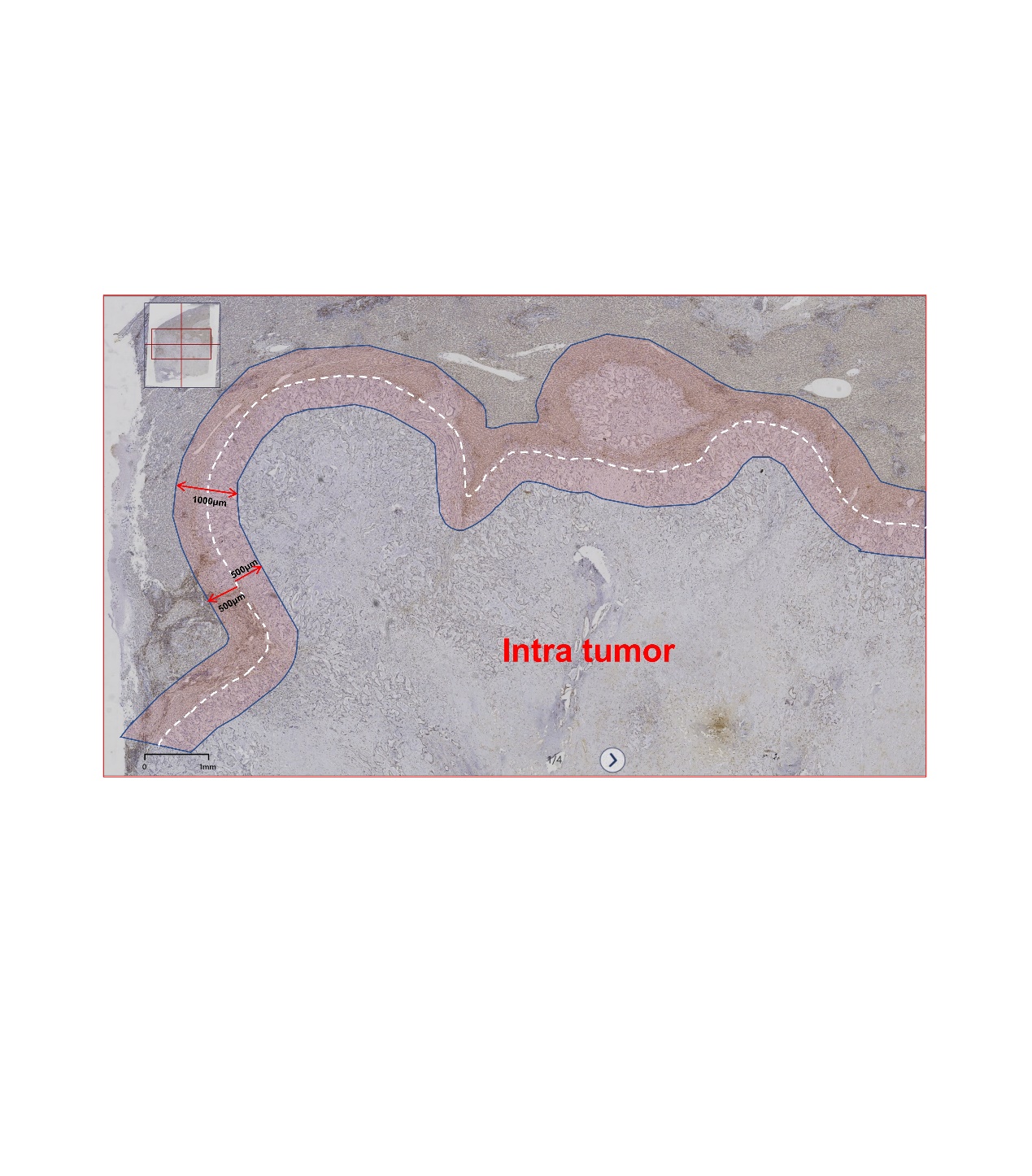
**

**Figure S6.**  Panoramic scan of the section of a tumor with liver invasion. The white dotted line marks the line of liver invasion and the red area of 1000μm width is defined as the Hepatic invasion margin.

**Figure S7**

**
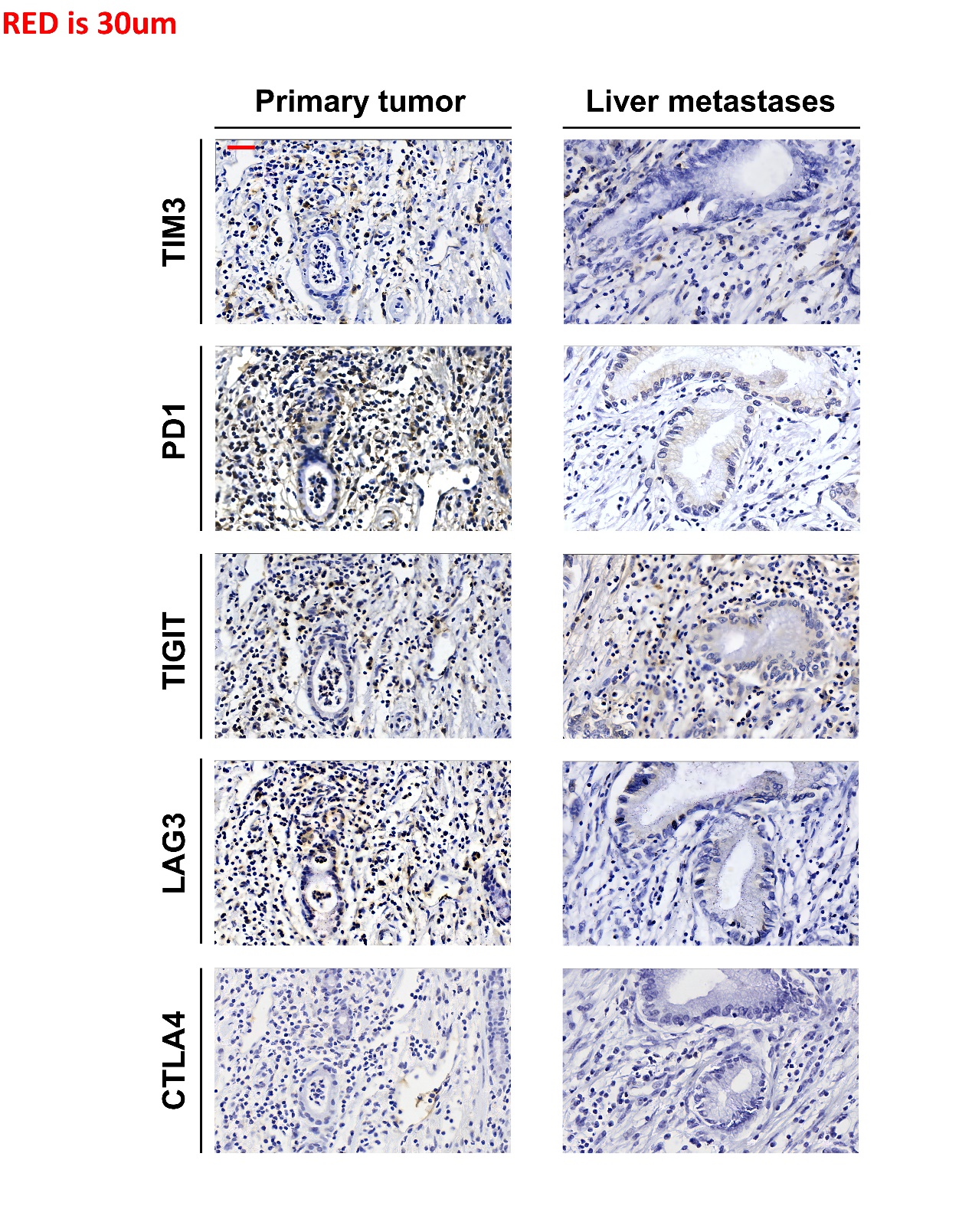
**

**Figure S7.**  Representative IHC images of PD1, TIM3, TIGIT, LAG3, CTLA4 staining are shown from the same GBC patient who had primary tumor (left column) and liver metastases (right column).

**Figure S8**


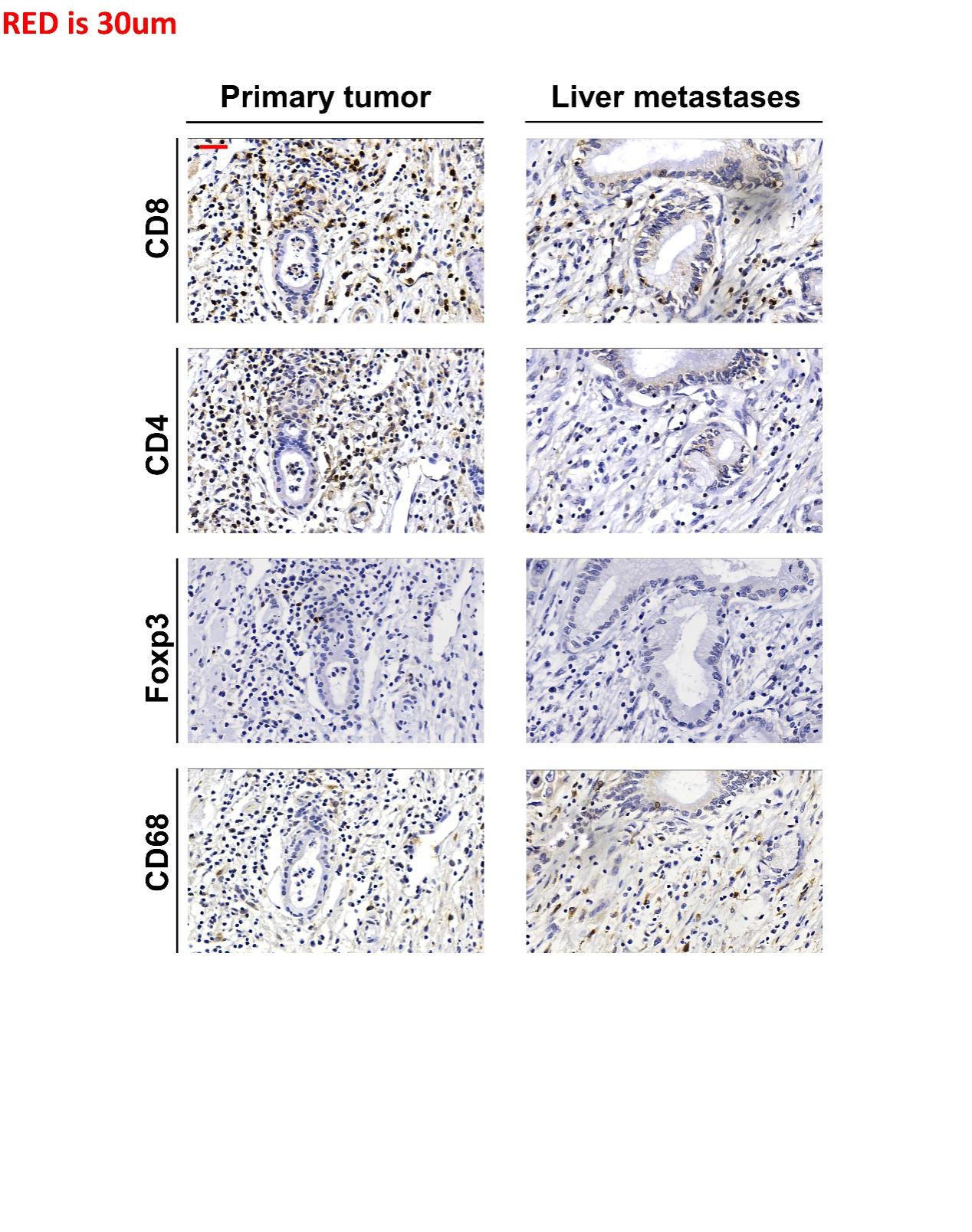


**Figure S8.**  Representative IHC images of CD8, CD4, CD68, Foxp3 staining are shown from the same GBC patient who had primary tumor (left column) and liver metastases (right column).

**Figure S9**


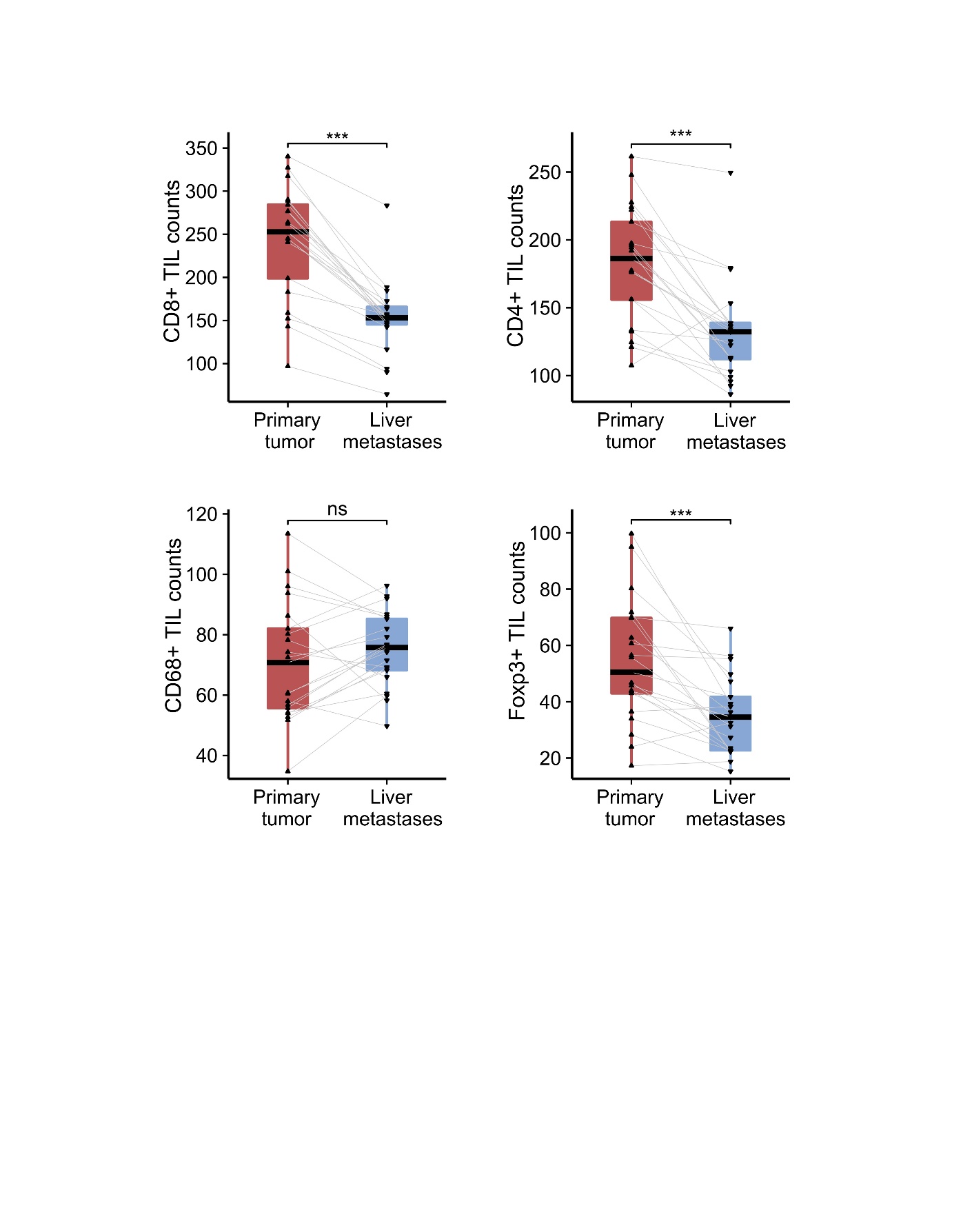


**Figure S9.**  Comparison of Immune cell counts between primary tumor and liver metastases in GBC (****P* < 0.001; ns: no significance).
