# Supplementary material for: Increased co-expression of PD1 and TIM3 is associated with poor prognosis and immune microenvironment heterogeneity in gallbladder cancer
Source: J Transl Med. 2023 Oct 12;21:717. doi: 10.1186/s12967-023-04589-3 (PMC10571407; doi:10.1186/s12967-023-04589-3)
Supplement: Supplementary file 2 — Image Analysis Protocol Detailed method for QuPath image analysis of immune. Detailed method for QuPath image analysis of immune. Detailed method for INFO image analysis of distinguishing. [file 12967_2023_4589_MOESM2_ESM.docx]

**Protocol Note 1. Detailed method for QuPath image analysis of immune**

**checkpoints expression ……………..……………………………………………….……1**

**Protocol Note 2. Detailed method for QuPath image analysis of immune**

**cells density …………………………………………………………................…………4**

**Protocol Note 3. Detailed method for INFO image analysis of distinguishing**

**cell subgroups ....................................................................................9**

**Note 1. Detailed method for QuPath image analysis of immune checkpoints expression**

1. Import whole slide image file to QuPath software

Whole slide image formats: Aperio (.svs, .tif), Hamamatsu (.vms, .vmu, .ndpi), Leica (.scn), MIRAX (.mrxs), Philips (.tiff), Sakura (.svslide), Trestle (.tif), Ventana (.bif, .tif), Generic tiled TIFF (.tif), Perkin Elmer (.qptiff), ImageJ, TIFF, JPEG, PNG

1. Set image type → ***Bright-field H-DAB*** → ***Apply***
2. Utilizing the ***magic wand annotation tool*** (
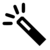
 ), regions of interest within each complete tumor tissue section are unmistakably delineated. Areas featuring tumor necrotic tissue, section folds, and extensive entrapped non-tumor tissue are prudently eliminated.


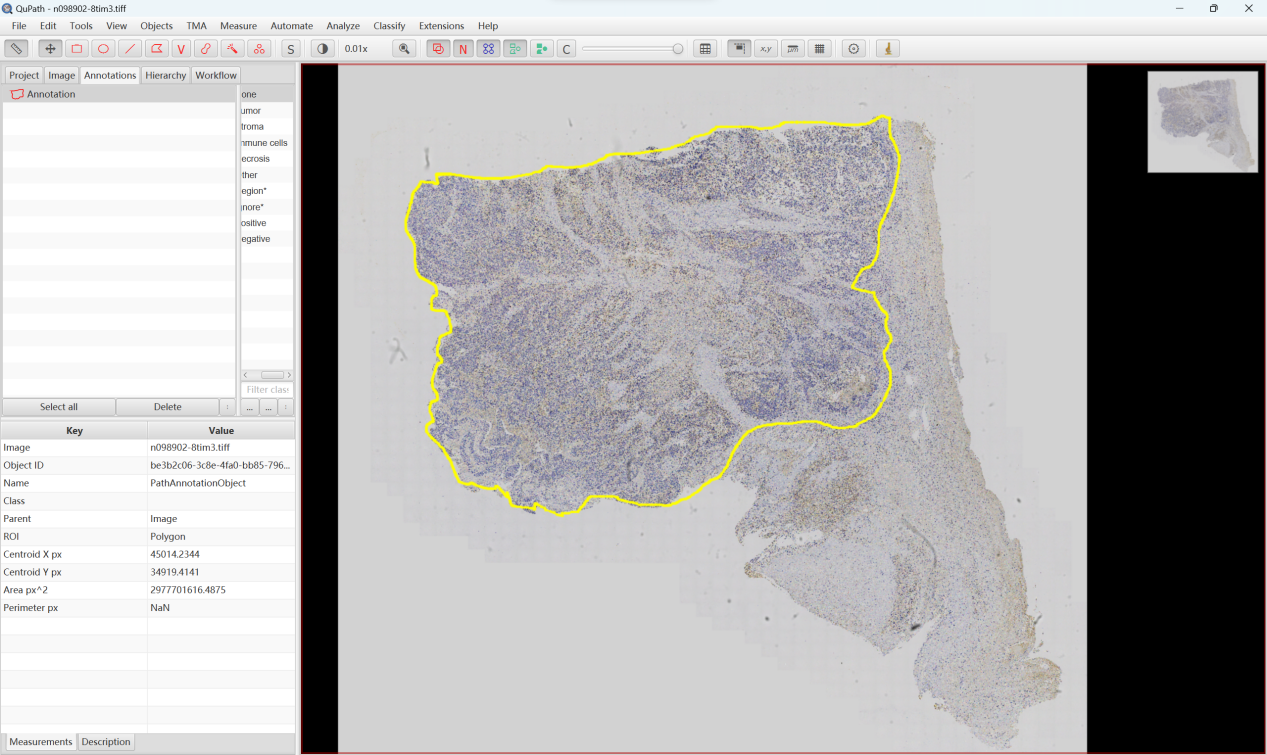


1. It is recommended to refine the stain estimates for each new batch of images, as a way to ensure consistent intensity of IHC staining. Draw an annotation with rectangle tool around a region containing examples of each stain and background (white space).

Then, select Analyze → Preprocessing → Estimate stain vectors → select Yes on pop up window → select OK on Visual Stain Editor pop up screen.

If there is a significant discrepancy between the background values of the plotted region and the original values, QuPath will prompt you to update the stored values. Confirm with 'Yes' only if the plotted region includes a representative background region. Continue by selecting 'Auto', check the revised colouring vectors and confirm with 'OK'. Finally, assign a unique identifier to the updated colour vector.

1. Perform Positive cell detection with single threshold

(From the tool bar, select Analyze → Cell detection → Positive cell detection → Set parameters as follows：

Detection image: Optical density sum

Check ‘Use opening by reconstruction’

Background radius: 8 µm

Median filter radius: 0 µm

Sigma: 1.5 µm

Minimum area: 10 µm2

Maximum area: 400 µm2

Intensity threshold: 0.1

Max background intensity: 2

Check ‘Split by shape’

Unselect ‘Exclude DAB (membrane staining)’

Cell expansion: 5 µm

Check ‘Include cell nucleus’

Check ‘Smooth boundaries’

Check ‘Make measurements’

Score compartment: Cell: DAB OD mean

Threshold 1+ : 0.2

Threshold 2+ : 0.4

Threshold 3+ : 0.6

Unselect ‘Single threshold’

Then go to the ‘RUN’ button. This will take a while.


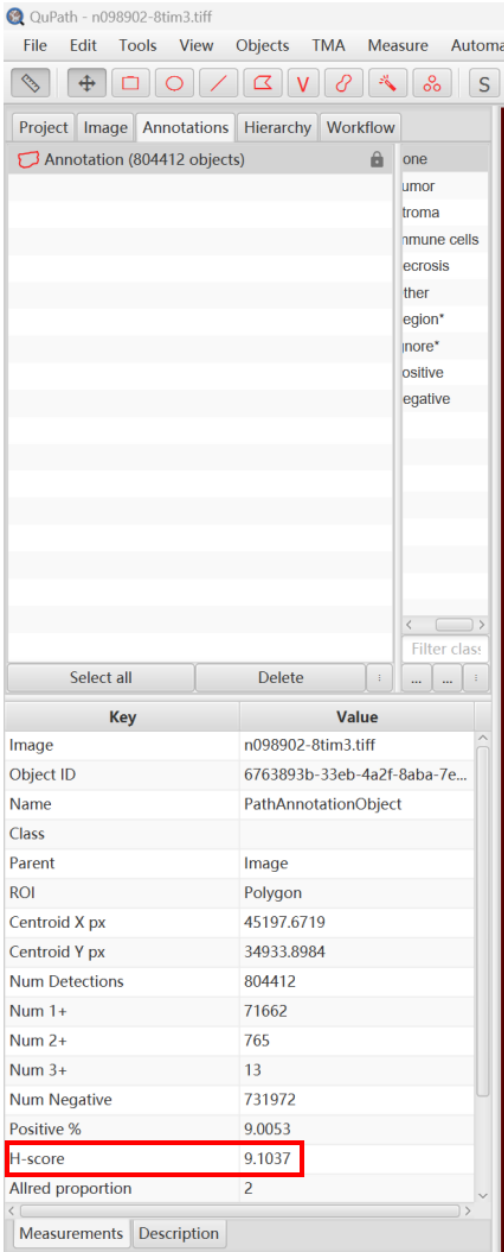


***The immune checkpoint expression***

***H score = ((1*Num^1+^ + 2*Num^2+^ + 3*Num^3+^) / Num detections ) * 100***

**Note 2. Detailed method for QuPath image analysis of immune cells density**

1. Import whole slide image file to QuPath software

Whole slide image formats: Aperio (.svs, .tif), Hamamatsu (.vms, .vmu, .ndpi), Leica (.scn), MIRAX (.mrxs), Philips (.tiff), Sakura (.svslide), Trestle (.tif), Ventana (.bif, .tif), Generic tiled TIFF (.tif), Perkin Elmer (.qptiff), ImageJ, TIFF, JPEG, PNG

1. Set image type → ***Bright-field H-DAB*** → ***Apply***
2. Utilizing the ***magic wand annotation tool*** (
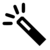
 ), regions of interest within each complete tumor tissue section are unmistakably delineated. Areas featuring tumor necrotic tissue, section folds, and extensive entrapped non-tumor tissue are prudently eliminated.


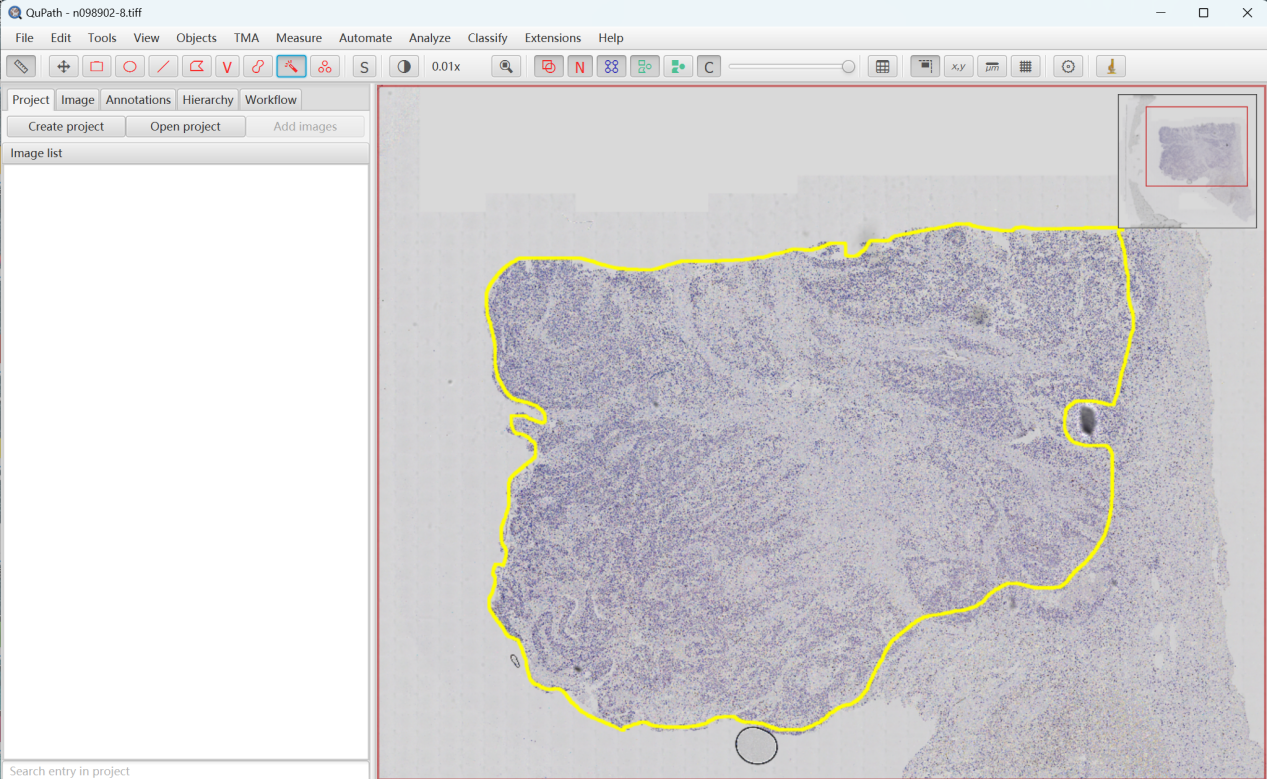


1. It is recommended to refine the stain estimates for each new batch of images, as a way to ensure consistent intensity of IHC staining. Draw an annotation with rectangle tool around a region containing examples of each stain and background (white space).

Then, select ***Analyze → Preprocessing → Estimate stain vectors → select Yes on pop up window → select OK on Visual Stain Editor pop up screen.***

If there is a significant discrepancy between the background values of the plotted region and the original values, QuPath will prompt you to update the stored values. Confirm with '***Yes***' only if the plotted region includes a representative background region. Continue by selecting '***Auto***', check the revised colouring vectors and confirm with '***OK***'. Finally, assign a unique identifier to the updated colour vector.


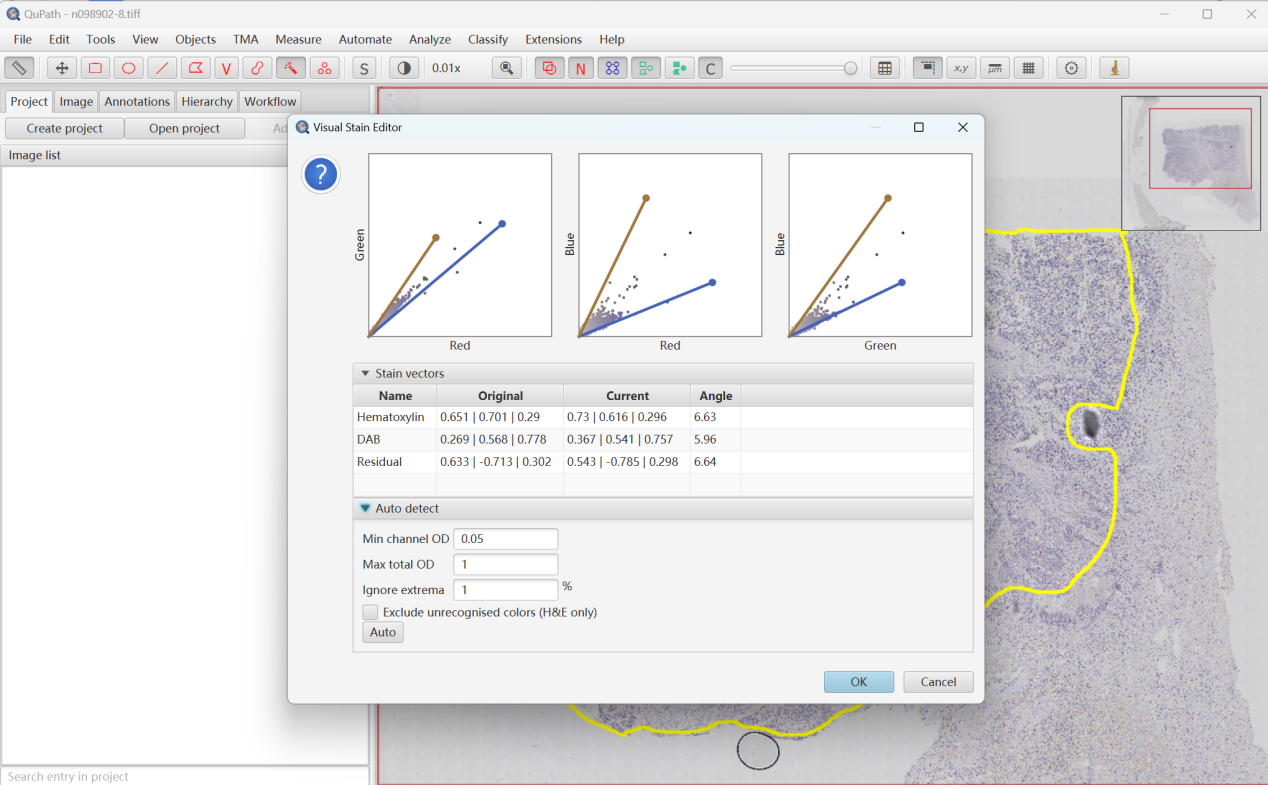


1. Create a grid with size ***400μm x 400μm***

(Region identification → From tool bar select ***Analyze → Tiles ＆ superpixels → Create tiles → Tile size 400μm → Trim to ROI: true → Make annotation tiles: true → Remove parent annotation: false → Run***)


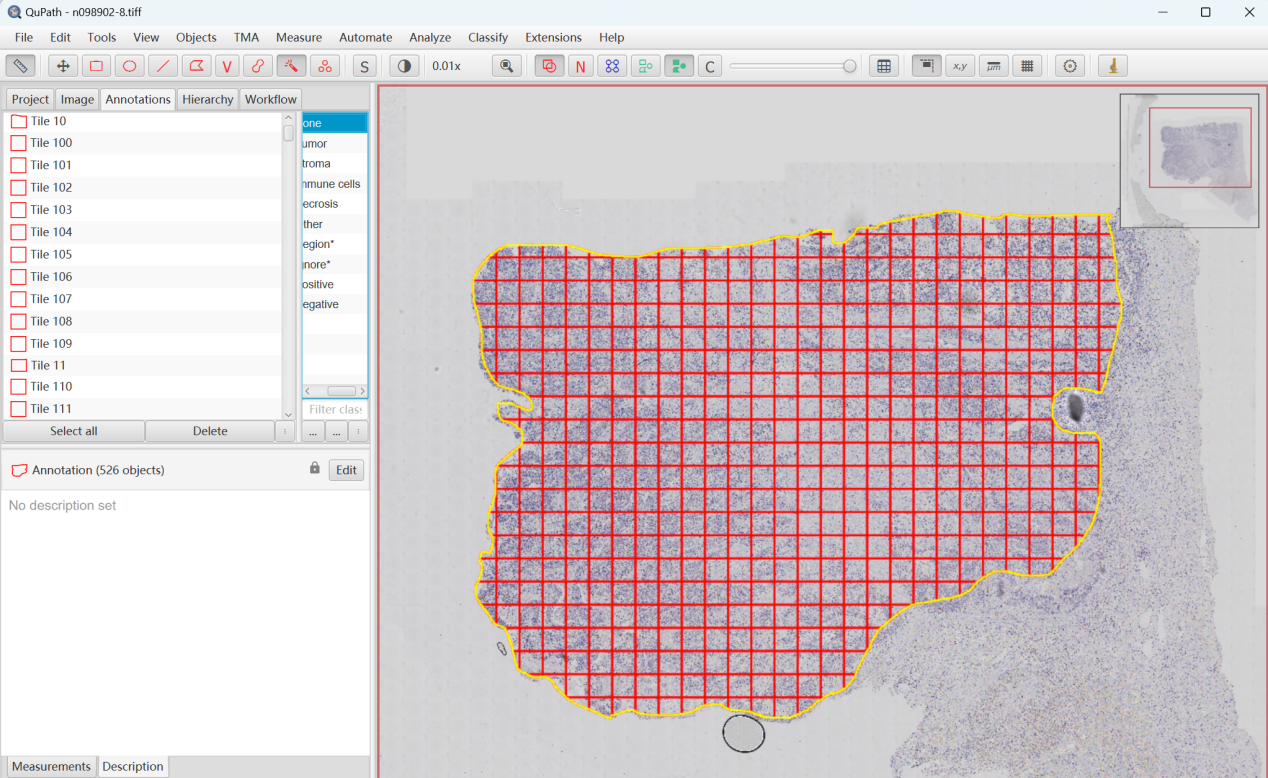


1. Select ***all annotations***, as these originate from the area delineated in step three.


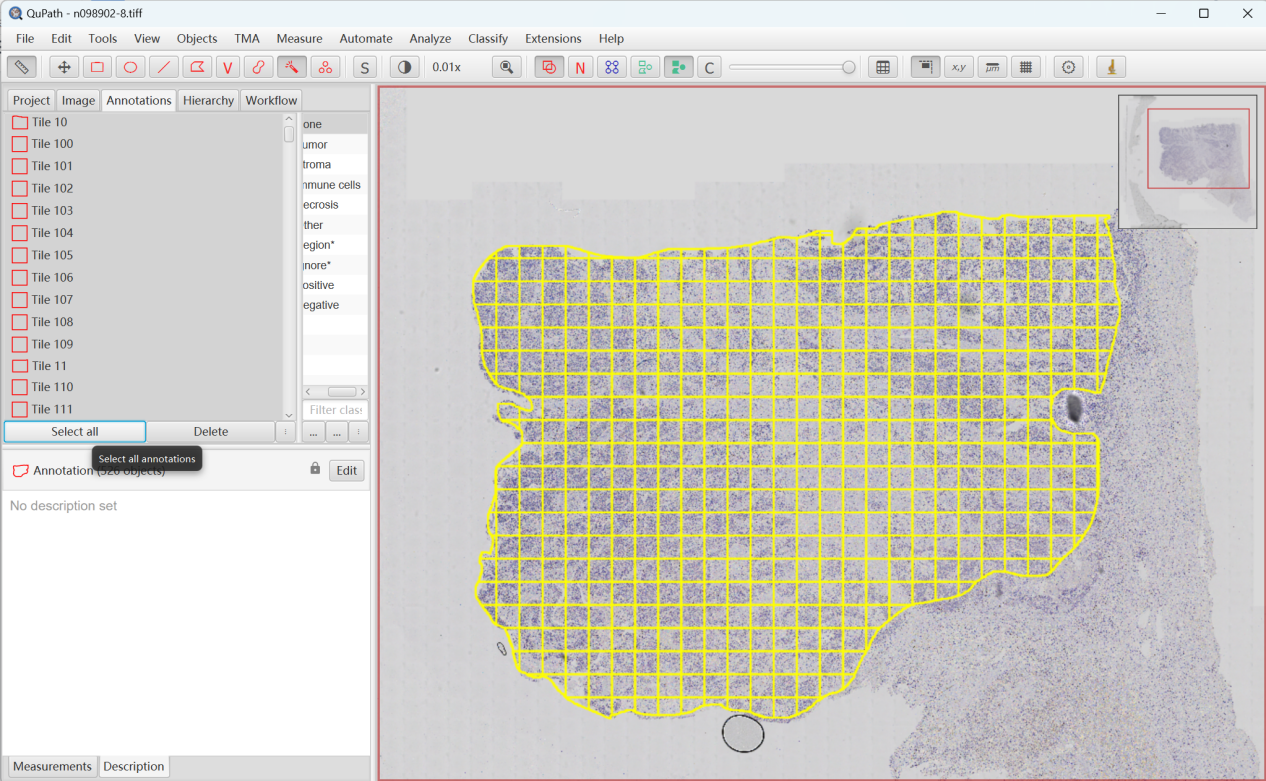


1. Perform Positive cell detection with single threshold

(From the tool bar, select ***Analyze → Cell detection → Positive cell detection → Set parameters as follows***：

Detection image: Optical density sum

Check ‘Use opening by reconstruction’

Background radius: 8 µm

Median filter radius: 0 µm

Sigma: 1.5 µm

Minimum area: 10 µm^2^

Maximum area: 400 µm^2^

Intensity threshold: 0.1

Max background intensity: 2

Check ‘Split by shape’

Unselect ‘Exclude DAB (membrane staining)’

Cell expansion: 5 µm

Check ‘Include cell nucleus’

Check ‘Smooth boundaries’

Check ‘Make measurements’

Score compartment: Cell: DAB OD mean

Check ‘Single threshold’

Then go to the ‘***RUN***’ button. This will take a while.


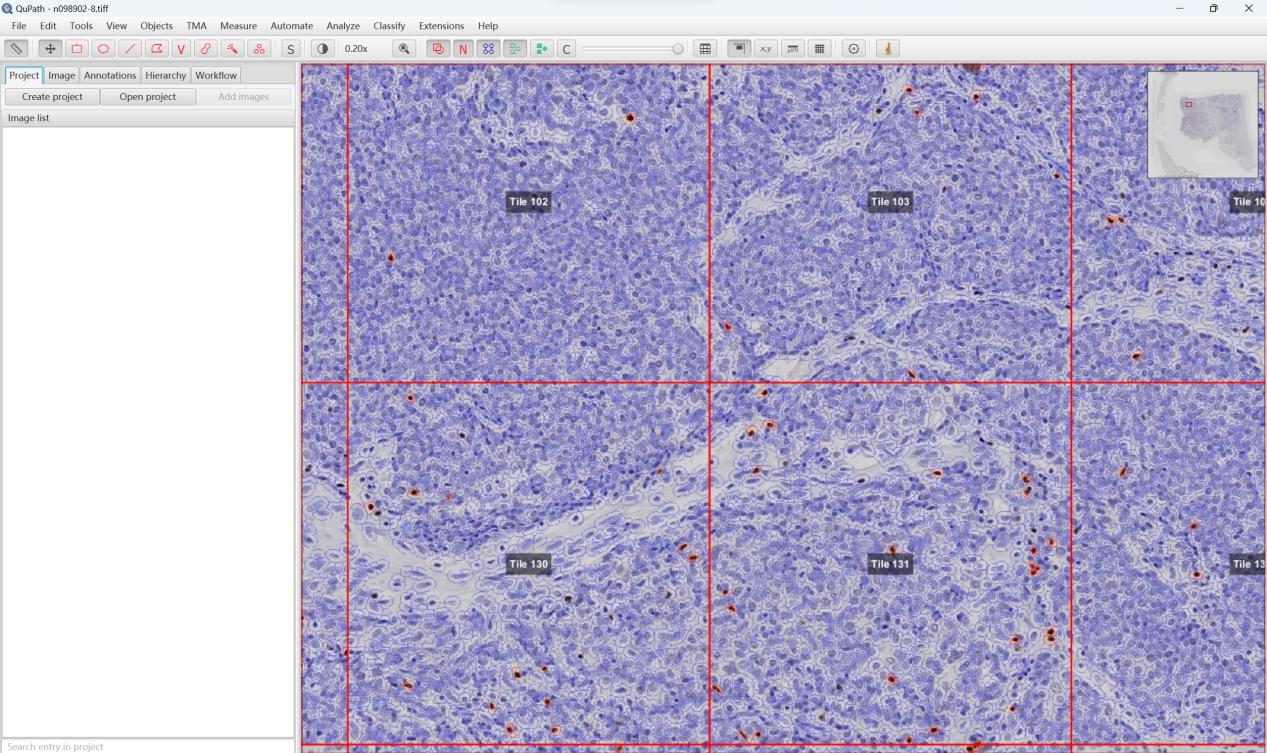


1. Then, from tool bar select ***Measure → Show annotation measurements***.

The counts for the positive cells across all annotations are listed as depicted in the illustration, with the counts for positive cells of the ***top four highest-ranked regions*** selected for further data analysis. We named these four regions as ***hot-spot areas.***


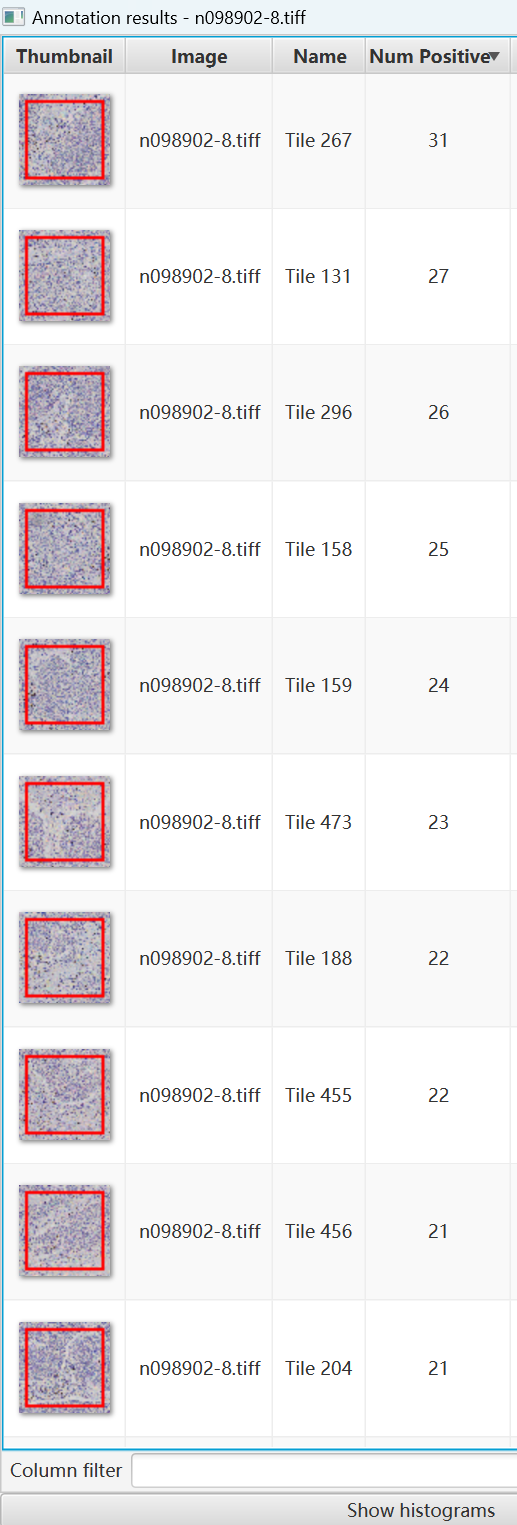


1. ***The definitive IHC cell count data*** derives from the mean value of the four ***hot-spot areas.***

**Note 3. Detailed method for INFO image analysis of distinguishing cell subgroups**

1. **Spectral splitting**

Select the fluorescent dyes, check all the fluorescent dyes used in the staining in the pop-up window, and click OK;

Edit the color of each fluorescent dye and the corresponding protein.

1. **Tissue segmentation**

Click on the dots behind the corresponding areas to circle them in the image. corresponding feature area in the image.

Click on “**Train Tissue Segmenter”** and wait for the software to learn automatically with an accuracy of 90% or higher

1. **Cell segmentation**

Check the cell structures to be recognized;

Adjust the Adjust the following parameters to make the cell separation more accurate;

a) Signal Intensity Threshold; 0.6

b) Splitting sensitivity; 0.9

c) Minimum nuclear size; 5

d) Cytoplasm thickness; 5

E) Membrane search distance; 5

1. **Cell phenotype**

① Tap Add to create a new cell type;

② Tap this icon to left-click the cell nucleus position on the picture and define the cell as a certain type;

We aim to screen the following eight cell subgroups.

PD1+TIM3+CD8+

PD1+TIM3-CD8+

PD1-TIM3+CD8+

PD1-TIM3-CD8+

PD1+TIM3+Foxp3+

PD1+TIM3-Foxp3+

PD1-TIM3+Foxp3+

PD1-TIM3-Foxp3+

③The number of all selected cells will be displayed here, and the number of cells of each type should be not less than 5, we choose 25 or more cells of each phenotype for optimal results;

④ Click Train Classifier, the software will learn according to the selected feature cells and categorize all cells;

⑤ Process the current image/process all images and go to the next step;

⑥The icon can show/hide the cell type split layer, or show only the cell type checked.

1. **Export data**
